# A Bis(Acridino)-Crown Ether for Recognizing Oligoamines in Spermine Biosynthesis

**DOI:** 10.3390/molecules29184390

**Published:** 2024-09-15

**Authors:** Péter Kisfaludi, Sára Spátay, Marcell Krekó, Panna Vezse, Tünde Tóth, Péter Huszthy, Ádám Golcs

**Affiliations:** 1 Department of Organic Chemistry and Technology, Budapest University of Technology and Economics, Szent Gellért Square 4, 1111 Budapest, Hungary; kisfaludi.peti@edu.bme.hu (P.K.); spatay.sara@edu.bme.hu (S.S.); panna.vezse@edu.bme.hu (P.V.); toth.tunde@vbk.bme.hu (T.T.); 2 Department of Pharmaceutical Chemistry, Semmelweis University, Hőgyes Endre Street 9, 1092 Budapest, Hungary; kreko.marcell@semmelweis.hu; 3 Center for Pharmacology and Drug Research & Development, Department of Pharmaceutical Chemistry, Semmelweis University, Üllői Street 26, 1085 Budapest, Hungary; 4 HUN-REN Centre for Energy Research, Konkoly-Thege Miklós Street 29-33, 1121 Budapest, Hungary

**Keywords:** biogenic oligoamines, spermine, spermidine, fluorescence, chemosensor, crown ether

## Abstract

Oligoamines in cellular metabolism carry extremely diverse biological functions (i.e., regulating Ca^2+^-influx, neuronal nitric oxide synthase, membrane potential, Na^+^, K^+^-ATPase activity in synaptosomes, etc.). Furthermore, they also act as longevity agents and have a determinative role in autophagy, cell growth, proliferation, and death, while oligoamines dysregulation is a key in a variety of cancers. However, many of their mechanisms of actions have just begun to be understood. In addition to the numerous biosensing methods, only a very few simple small molecule-based tests are available for their selective but reversible tracking or fluorescent labeling. Motivated by this, we present herein a new fluorescent bis(acridino)-crown ether as a sensor molecule for biogenic oligoamines. The sensor molecule can selectively distinguish oligoamines from aliphatic mono- and diamino-analogues, while showing a reversible 1:2 (host:guest) complexation with a stepwise binding process accompanied by a turn-on fluorescence response. Both computational simulations on molecular docking and regression methods on titration experiments were carried out to reveal the oligoamine-recognition properties of the sensor molecule. The new fluorescent chemosensor molecule has a high potential for molecular-level functional studies on the oligoamine systems in cell processes (cellular uptake, transport, progression in cancers, etc.).

## 1. Introduction

**Figure 1:**
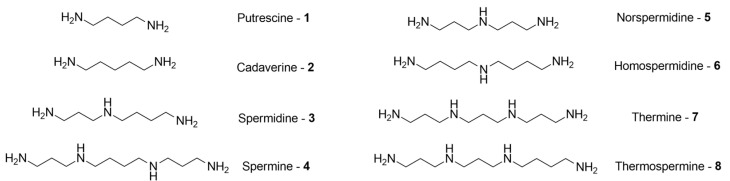
Examples for important aliphatic oligoamines (**1–8**).

A great number of biologically active molecules contain at least one or more amino functional groups. Biogenic amines are found in various organisms, cells, foods, and beverages and can be classified as monoamines, oligoamines, and polyamines depending on the number of amine functions they contain. They are typically synthesized by living organisms through enzymatic decarboxylation of amino acids [1,2,3]. Biogenic aliphatic oligoamines such as spermine (**4**), spermidine (**3**), putrescine (**1**), and cadaverine (**2**) (Figure 1) are among the most important ones, which can be found in almost all living organisms. They exist within cells in protonated forms and play important roles in diverse biological processes, such as cell growth, gene regulation, nucleic acid stabilization, and cell proliferation [4,5,6].

In vertebrates, two oligoamines, spermidine (**3**) and spermine (**4**), are present along with their precursor, the diamine putrescine (**1**). The absence of a normal oligoamine content can have a significant impact on cell functions including differentiation, apoptosis, motility, and resistance to oxidative and other stresses [7,8]. The importance of these oligoamines is shown not only in the effects of their absence but also in their extensive spectrum of applications. It has been shown that elevated spermine (**4**) levels can be used to indicate the presence of cancer cells, making it possible to use spermine (**4**) as a cancer biomarker [9]. Furthermore, the high concentration of spermine (**4**) and spermidine (**3**) can also be connected to psoriasis, where patients have about 5.5 times the amount of these oligoamines than healthy people [10]. On the other hand, concentrations below the normal amount can be linked to aging related illnesses, such as *Parkinson*’s disease [11]. Studies have also shown that spermidine (**3**) holds promise for both diagnosis and treatment of ocular diseases [12].

The relationship between oligoamines and both cancer and essential cellular processes has stimulated the development of methods for tracking oligoamine-mediated biological processes. Given that most tumors exhibit elevated activity in the oligoamine transport [13], chemical probes designed to exploit this system could be used to target cancer cells for both imaging and chemotherapy [14]. Typically, studying oligoamine transport in cells relies on the use of radiolabeled (^3^H or ^14^C) or fluorescently labeled oligoamines. Recently, fluorescent oligoamine conjugates incorporating a symmetrical BODIPY fluorophore have been reported and used to monitor oligoamine uptake by cancer cells [15]. The simple synthesis of these clickable oligoamine live-cell probes facilitates their commercial viability, making the tracking of oligoamine-mediated processes more accessible. The biological role of spermine (**4**) and spermidine (**3**) is still a dynamically researched area, with new discoveries being made even in recent years. It has been shown that spermidine (**3**) plays a role in both aging and aging related illnesses [16]. Spermidine (**3**) level decreases with age, but supplementation can improve or postpone various age-related conditions, including those impacting the immune system, through directly affecting T cell function and ATP production. Due to these properties, it shows promising antitumor effects [17]. In the case of spermine (**4**), a study in 2024 suggested that the elevated levels of spermine (**4**) in the brains of *Alzheimer*’s disease patients indicate its involvement in the disease and highlight its potential as a therapeutic target [18]. Along with their importance in human health, these oligoamines can also be used in the agricultural sector to enhance seed germination and seedling performance [19]. 

Today, mostly traditional chromatographic methods are used for the identification and quantification of spermine (**4**) and spermidine (**3**) [2]; among them, the most used techniques are capillary electrophoresis (CE) and high-performance liquid chromatography (HPLC) for their small sample requirement and relatively low detection limit [20,21,22]. However, chromatographic methods require extensive sample preparation including solid-phase extraction and matrix solid-phase dispersion of the biogenic amines [23]. Instrumental-based analytics also require the use of complex and expensive devices, making them less accessible and useful in some routine diagnostics. Chemosensors provide a fast and straightforward alternative for the detection of the biogenic amines, eliminating the need of specified instrumentation. They can be used to monitor biological samples, such as blood, saliva, and urine reducing the need for invasive procedures. Although detecting substances present in bio-fluids can be crucial for investigating various diseases and illnesses (such as predicting prostate cancer based on spermine (**4**) levels in urine [24]), this task is complicated by the presence of hundreds of molecules, often with similar properties, appearing in both trace and micromolar amounts [25]. Investigating bio-fluids with such complex molecular compositions using classical methods poses a challenge, but chemosensors’ versatility enables selective examination of specific substances of interest without any need of pretreatment procedure. Furthermore, chemosensors provide real-time analysis enabling continuous monitoring of biological processes. They can also be used for bioimaging [26], capturing cellular-level photographs of biological indicators, organs, tissues, etc.

These sensors are based on molecular recognition and generate a signal in the presence of an analyte [25]. The molecule used as a chemosensor must have chromogenic or fluorogenic properties, which change upon the complexation of the analyte. This change in the optical properties enables a straightforward detection [27,28]. Most chemosensors use the “binding site-signaling site” approach, where separate units provide these properties. In our method, these properties are in the same place by incorporating the fluorophore unit as a part of the binding cavity. This fluorophore can alter its emission through a direct internal charge transfer (ICT) process upon complexation, offering numerous advantages over other methods, such as fast response, high sensitivity, and easy regulation [29]. 

The research and development of chromogenic and fluorogenic chemosensors for detecting biologically active molecules have become an increasingly prominent area in recent years, especially in the cases of those lacking useful UV–vis spectral signatures. Several articles report on new chemosensors for detecting biogenic amines by utilizing the interactions of small molecular chromophores and fluorophores [30,31,32,33,34], organic polymers [35], coordination compounds [36], and nanoparticles [37]. Selective detection of aliphatic biogenic amines is complicated by their high structural similarity. In the case of protonated oligoamines, they contain several positive centers, which can serve as potential binding sites. Their aliphatic nature results in high conformational flexibility, making the increase in entropy difficult during coordination. Due to these difficulties, many chemosensors exhibit limited ability to distinguish between spermine (**4**), spermidine (**3**), cadaverine (**2**), and putrescine (**1**) [33,38]. To our knowledge, there is only one small fluorescent chemosensor molecule, which can selectively detect spermine (**4**) with little to no response from other analogues [34]. The use of small chemosensor molecules is advantageous, because their selectivity for specific analytes can easily be adjusted [39]. They are generally inexpensive to produce, tolerant to varying conditions, while interactions in biological environments are minimal. Moreover, their small molecular size facilitates easy integration into samples.

Compounds developed in recent years and capable of chemosensor-based oligoamine analysis often include carboxyl groups, which interact with amines through hydrogen bonding or electrostatic interactions [40]. Previously used compounds include carboxylic acid-functionalized poly(thiophenes), which employ a colorimetric method for the detection and identification of amines [41]. Additionally, tetraphenylethenes with carboxyl groups have been used; these are aggregation-induced emission active molecules, which remain non-emissive in solution but exhibit intense fluorescence when aggregated or in the solid state. This is due to the restriction of intramolecular rotations, which prevents nonradiative deactivation and fluorescence quenching [32,33]. A dicarboxylated ethynylarene was also used for the detection of oligoamines by its carboxylic acid subunits, demonstrating a turn-on fluorescence signal [39]. Other methods include the use of small-molecule amphiphiles and function by aggregation, such as fluorescent dyes like trisodium-8-hydroxypyrene-1,3,6-trisulfonate, which can be converted into a frustrated amphiphile upon alkylation [34]. It was found by *Köstereli* et al. that a turn-off response is demonstrated by this amphiphile in the presence of spermine (**4**), even at nanomolar concentrations, making it an effective spermine-selective chemosensor. A similar method was developed by *Singh* and coworkers using a perylenediimid derivative as the fluorescent dye, achieving a turn-on response [42].

Macrocycles with an 18-crown-6 ether-type cavity are able to complex both protonated and neutral primary amines (with the complex stability being weaker in the case of neutral ones) by forming hydrogen bonds with the nucleophilic heteroatoms of the host [43,44]. Attributed to these properties, crown ethers have been found suitable for several protonated amine-selective applications, for example the separation of ammonium salts from byproducts [45], the extraction of biogenic amines [46], and the separation of both chiral and non-chiral amino acids [47]. The achievements of crown ethers, in the selective separation of amine derivatives based on molecular recognition, suggest that these macrocycles could serve as effective chemosensors for the detection and selective separation of biogenic amines [48]. The difference in the number of amine functions, and chain lengths of the oligoamines can be utilized, if crown ethers containing two macrocycle units are used, making a multiple-point interaction to take place. In such cases, aside from the hydrogen bonds formed by the two macrocycle rings, other interactions (for example cation*-π* interaction) can also take place with the heterocyclic subunits, thereby enhancing the overall stability of the complex.

**Figure 2:**
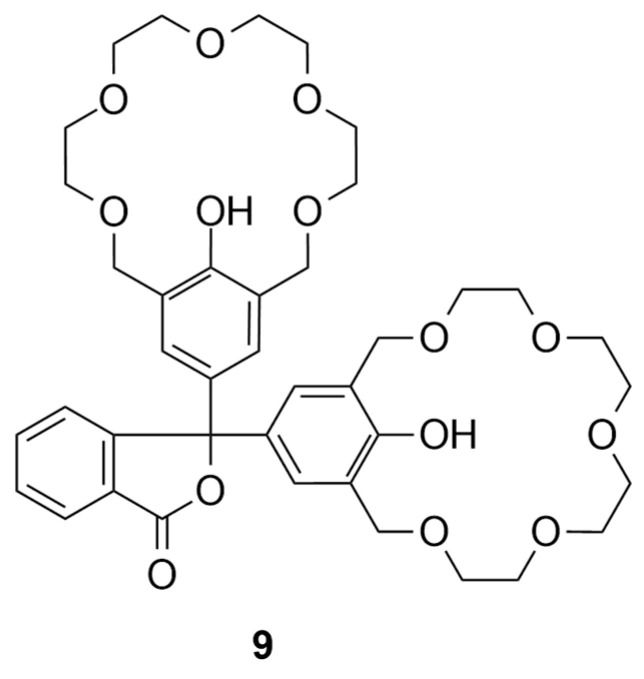
The host molecule used by *Tanima* and coworkers.

Such crown ether has been used before as a chemosensor for the detection of spermine analogues, based on colorimetry reported by *Tanima* and coworkers [30]. This chemosensor (**9**, see Figure 2) is the closest one, which we report here. To our knowledge, no other method utilizes molecules containing two crown ether units for this purpose.

A phenolphthalein derivative with two crown ether units (**9**) was used by *Tanima* and coworkers, which was able to bind spermidine (**3**) and spermine (**4**) by a three-podal interaction. With structural modifications of host molecule **9**, the colorimetric response was also transformed into a fluorescent one. The host molecules demonstrated a turn-off response, which is less suitable for distinguishing between response quenching and background interference. In contrast, turn-on fluorescence response provides a clearer and more definitive signal. Great selectivity for the “naked eye” recognition of spermine (**4**) and spermidine (**3**) was achieved over the other biogenic amines, while the host remained inactive against tryptamine (10 eq.) and cadaverine (**2**) (10 eq.), although showing the color response after spermine (**4**) was added (<1 eq.) to these interfering amines. However, a clear distinction between spermine (**4**) and spermidine (**3**) was not achieved, as the binding constants (log*K*) were close. For spermidine (**3**), the log*K* values ranged from 2.9 to 3.2, and for spermine (**4**), they ranged from 3.4 to 3.6, depending on the host used. 

## 2. Results and Discussion

### 2.1. Molecular Design

**Figure 3:**
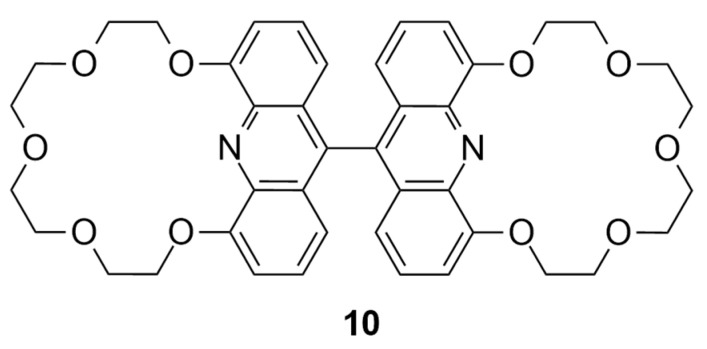
Structure of the new bis(crown ether) host molecule.

We designed bis(acridino)-crown ether **10** (Figure 3), which functions as a sensitive chemosensor for protonated aliphatic biogenic oligoamines and can selectively differentiate between short- and long-chained ones.

Host molecule **10** has two 18-crown-6 ether-type cavities with sizes, which match those of the protonated primary amino groups (*r* = 1.4 Å and *r* = 1.42 Å, respectively) [43,44]. This allows stable coordination by hydrogen bonding between the electrophilic ammonium ion and nucleophilic heteroatoms of the host [49]. The molecule exhibits fluorescence due to its acridine units. Due to electron conjugation, filling even one cavity induces a fluorescent response in these units. It shows a turn-on fluorescent response in contrast to the host molecules used by *Tanima* and coworkers [30]. Although both have their advantages, turn-on fluorescence responses are typically preferred to turn-off ones. They amplify the fluorescence signal significantly upon binding the analyte, thereby increasing the sensitivity to detect it. This amplification not only reduces the detectable lowest concentration of the analyte, but also improves the background suppression by maintaining minimal baseline fluorescence during interaction with the analyte, minimizing interference from other substances in the sample [50].

Besides being a good fluorophore, the acridine unit provides structural rigidity due to its planar structure. With the two macrorings being connected through the acridine units, not only is the distance between the two macrocyclic rings optimal for the length of the spermine (**4**) chain without causing significant structural distortion, but they also fixate both macrorings due to their planarity. In this arrangement, if the guest molecule contains a protonated interchain amine function, the acridine units within the host molecule can further enhance the formation of the host–guest complex by an additional aromatic *π-*cation interaction. Furthermore, the nitrogen atom of the acridine unit shows a stronger affinity for hydrogen bonding compared to oxygen atoms, thereby enhancing selectivity toward protonated primary amines [49]. These combined features offer great sensitivity, selectivity, and overall detection capabilities in analytical and diagnostic applications, making host molecule **10** suitable for use as a chemosensor.

### 2.2. Synthesis of the New Host Molecule

Herein, we introduce two different procedures for the synthesis of host molecule **10** and demonstrate its use as a chemosensor by both analytical studies and computational chemistry simulations using several biogenic and non-biogenic mono- and oligoamines, including spermine (**4**) and spermidine (**3**). The synthesis of macrocycle **10** started with the commercially available and relatively cheap 3-methoxybenzoic acid, which gave 4,5-dimethoxyacridone (**11**) by a multistep procedure as reported [51]. 4,5-dimethoxyacridone (**11**) was converted to the appropriate chloro-acridine derivative (**12**) using phosphorus pentachloride in phosphoryl chloride according to a previously described method [52], but with an improved workup, in which most of the phosphoryl chloride was evaporated from the reaction mixture under reduced pressure prior to pouring it into the trimethylamine solution. This adjustment eliminated the need for the excessive amounts of trimethylamine and dichloromethane used in the original method and resulted in a purer product.

**Scheme 1:**
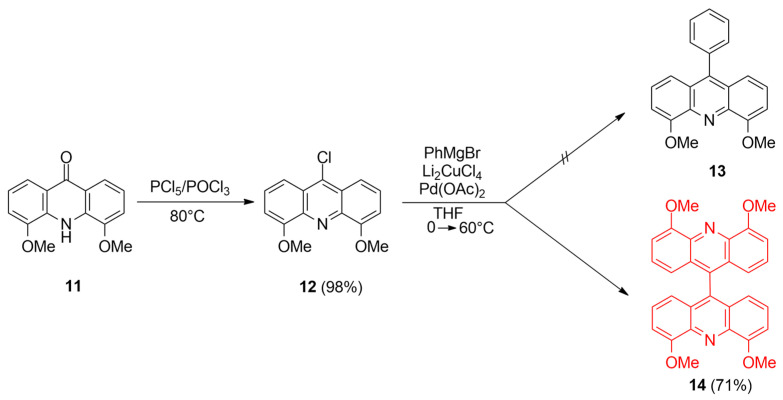
The synthetic procedures for the preparation of 9-chloro-4,5-dimethoxyacridine (**12**), a *Karasch* type coupling reaction, its intended product (**13**) and the byproduct (**14**) (the new compounds are indicated in red).

The chloro-acridine derivative (**12**) was then subjected to a *Kharasch* type cross-coupling reaction with dilithium tetrachlorocuprate and palladium acetate as catalysts, in the presence of phenylmagnesium bromide, where the intended product was 9-phenyl derivative **13** (Scheme 1).

**Scheme 2:**
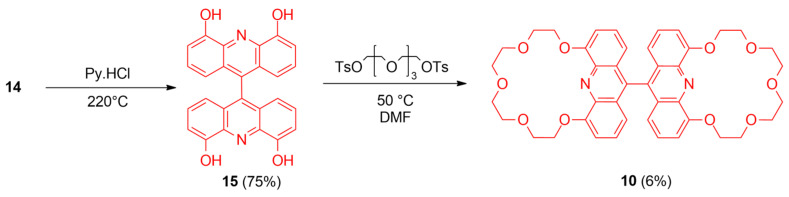
The procedure for obtaining bis(crown ether) **10**: demethylation of **14** followed by the macrocyclization (the new compounds are indicated in red).

Although the expected product could not be obtained, byproduct 4,4′,5,5′-tetramethoxy-9,9′-biacridine (**14**) was isolated with a good yield (71%). After identifying this byproduct, we determined that it would be used for the synthesis of macrocycle **10** (Scheme 2). The methyl groups of **14** were cleaved with pyridinium chloride to obtain tetrahydroxy-biacridine **15**. Macrocyclization of **15** with tetraethylene glycol ditosylate was carried out in DMF using potassium carbonate as a base to produce **10**. Bismacrocycle **10** was isolated with a low yield (6%), which is typical for these types of reactions, especially in this case where the macrocycle rings can be closed in several ways. The primary byproducts include compounds where the ditosylate reacted with hydroxyl groups on two different acridine subunits of the biacridine, as well as partially reacted compounds where only one ring has closed. Other minor byproducts include compounds where the ditosylate reacted with only one hydroxyl group, as well as those where the ditosylate reacted with hydroxyl groups on two different biacridine molecules, leading to no macrocyclic ring formation.

**Scheme 3:**
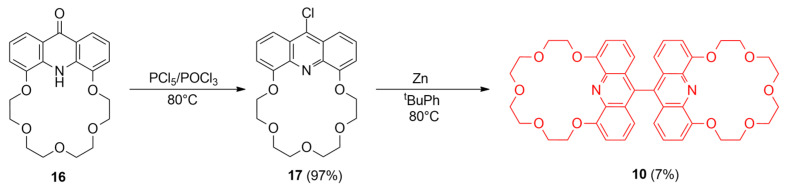
Optimized synthesis for the previously reported, but not isolated chloroacridino-crown ether **17**, and preparation of bis(crown ether) **10** by a coupling reaction (the new compounds are indicated in red).

Another method for the synthesis of macrocycle **10** is outlined in Scheme 3, where the starting material was a reported acridono-crown ether (**16**) [53]. Crown ether **16** reacted with phosphorus pentachloride in phosphoryl chloride resulting in chloroacridino-crown ether **17**, which is reported as an unstable compound and has not been isolated or characterized before [53]. Chloroacridino-crown ether **17** was converted to macrocycle **10** by a coupling reaction using zinc powder in *tert*-butylbenzene giving a 7% yield.

The probable explanation for the low yield of this reaction is that chloroacridino-crown ether **17** can form a complex with about four moles of water (according to the NMR spectrum of the crude product). Coordinating water within the cavity is a common phenomenon among analogous macrocycles [54]. In this case, decomplexation of water takes place during the reaction, which leads to the hydrolysis of **17** resulting in **16** as a byproduct. Attempts were made to decomplex water by passing **17** through a column filled with aluminum oxide, but one mole of water always remained in complex form even after multiple attempts, which decreased the yield of **10**.

### 2.3. In Silico Structure Optimization of Crown Ether—Oligoamine Complexes

Molecular modeling was used to gain insight into the nature of interactions between the host molecule and investigated oligoamines. The optimal structures of **10** and its complexes formed with spermine (**4**), spermidine (**3**), norspermidine (**5**) and *N*,*N*′-bis-(2-aminoethyl)propane-1,3-diamine (**19**) were determined by density functional theory (DFT) calculations.

**Figure 4:**
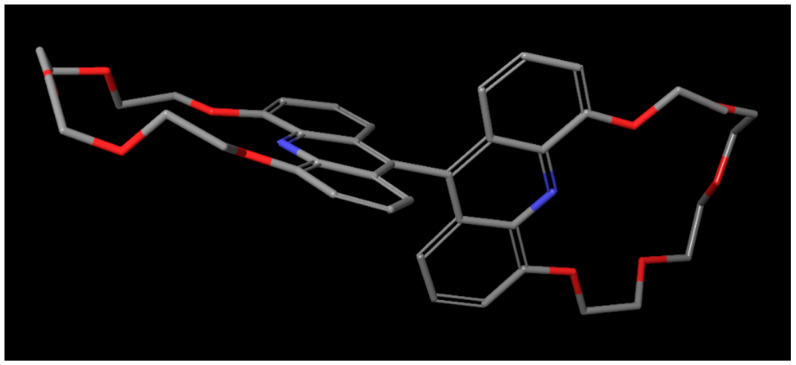
Optimal geometry determined for host molecule **10** using DFT. (Grey, red, and blue atoms represent *C*, *O*, and *N*, respectively).

In its minimal energy state, host molecule **10** assumes a fully extended conformation (Figure 4). The planarity of the acridine units is undisrupted and the centers of the crown ether macrorings stay in the proximity of the aromatic planes. The bond connecting the acridine units falls into both aromatic planes; therefore, bending cannot be observed between the two rings. The equilibrium conformation determined in the modeling of host molecule **10** closely aligns with the observed conformation of biacridines [55], with an angle of approximately 80 degrees between the planes of the two acridine units.

**Figure 5:**
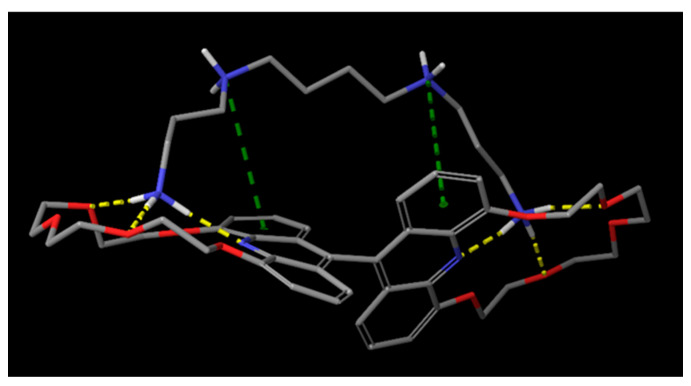
Optimal geometry determined for the complex of host molecule **10** with spermine (**4**) using DFT. (Grey, red, and blue atoms represent *C*, *O*, and *N*, while yellow and green dashed lines represent hydrogen bonds and cation-*π* interactions, respectively).

The optimized structure for bis(crown ether) **10** complex with spermine (**4**) (Figure 5) suggests a four-podal interaction. The crown ether macrorings coordinate the protonated primary amino groups of spermine (**4**), forming hydrogen bonds with the oxygens at the 7th and 13th positions and the nitrogen of the acridine units. The protonated secondary amine parts are in proximity to the acridine units, and both parts form cation–*π* interactions with the acridine units. According to the model, spermine (**4**) is ideal in terms of chain length, since **10** retains its extended conformation, where both crown ether rings lie in the aromatic planes of the acridine units. Torsion in the acridine units can be observed upon the coordination of spermine (**4**).

**Figure 6:**
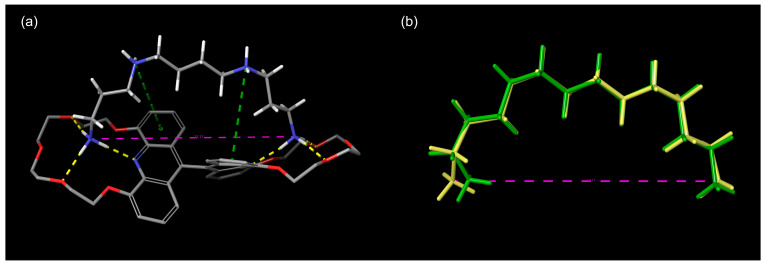
The distance between the terminal amines of spermine in complex with compound **10** indicated as purple dashed line: (**a**); overlay of the conformation of spermine bound to the host molecule **10** (yellow) and a local minimum energy state of spermine obtained using the “MacroModel Minimization” function of Schrödinger (green): (**b**).

A distance of 12.2 Å can be measured between the terminal amines of spermine in complex with compound **10** (Figure 6a). It is apparent that the geometry of bound spermine is very close to, and highly overlaps with, a minimal energy state conformation of the molecule, i.e., only a minuscule strain occurs in the structure of spermine upon binding to the host molecule **10** (Figure 6b).

**Figure 7:**
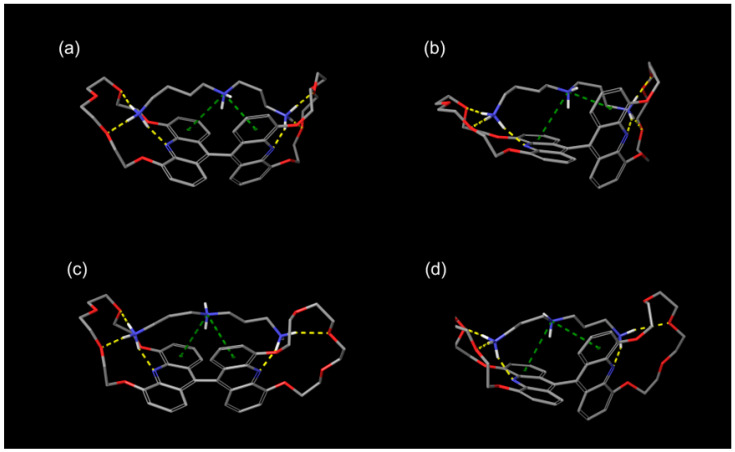
Optimal geometries rotated at different angles determined for complexes of host molecule **10** with spermidine (**3**): (**a**,**b**), and norspermidine (**5**): (**c**,**d**) using DFT. (Grey, red, and blue atoms represent *C*, *O,* and *N*, while yellow and green dashed lines represent hydrogen bonds and cation-*π* interactions, respectively).

In the complexes with optimal coordination determined for spermidine (**3**) and norspermidine (**5**) (Figure 7), the protonated secondary amine part forms cation–*π* interactions with both acridine units, while the protonated primary amino groups coordinate with the heteroatoms of the crown ether rings. Due to the shorter chain lengths, at least one of the two crown ether rings has to rotate out of the plane of the acridine unit in order to coordinate with the protonated primary amino groups. Both protonated primary amino groups of spermine (**4**) are ideally positioned for coordination with the crown ether rings; however, in our model, norspermidine (**5**) lacks one hydrogen bond with the heteroatoms of the crown ether rings, due to its shorter chain than the ideal distance for coordination. The acridine units show some degree of distortion and bending compared to the optimized structure of the uncomplexed host molecule.

**Figure 8:**
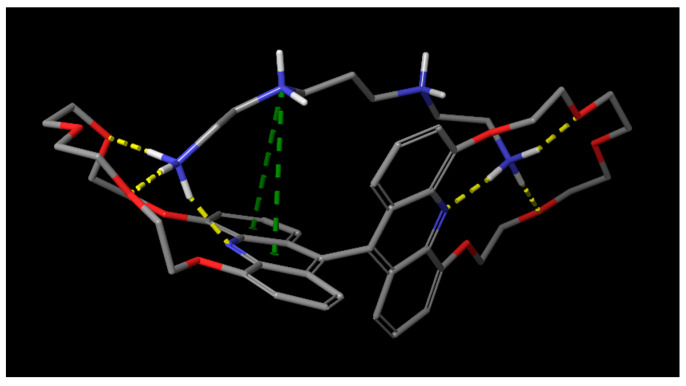
Optimal geometry determined for the complex of host molecule **10** with protonated *N*,*N*′-bis-(2-aminoethyl)propane-1,3-diamine (**19**) using DFT. (Grey, red and blue atoms represent *C*, *O* and *N*, while yellow and green dashed lines represent hydrogen bonds and cation-*π* interactions, respectively).

In the coordination complex determined for *N*,*N*′-bis-(2-aminoethyl)propane-1,3-diamine (**19**), the two protonated primary amino groups occupy the interiors of the crown ether rings, but only one cation–*π* interaction is present with one of the acridine units, although **19** has two protonated secondary amine parts (Figure 8).

The distance of the protonated amine parts is not ideal to form two cation–*π* interactions at the same time. Torsion of the acridine units can be observed, as well as considerable bending between the aromatic planes compared to those of uncomplexed biscrown ether **10** can also be seen.

### 2.4. Spectroscopic Studies on Molecular Recognition with Oligoamines

The molecular recognition of host molecule **10** with protonated oligoamines was studied by fluorescence spectrophotometric titrations. One of the aims was validating the complexation properties predicted by computational modeling. On the other hand, experimental results may reveal important differences and effects, which play crucial roles in practice, but cannot be predicted by computational chemistry. Moreover, the comparison of theoretical and experimental methods enables the extrapolation of established relationships to the range of potential guest molecules, which have not been investigated yet.

The titrations were carried out both in nonaqueous and semi-aqueous media, using acetonitrile and distilled water as solvents. (Fully aqueous medium could not be used due to the poor solubility of the host molecule in water.) The analyte solutions were added with a Hamilton-syringe to the 10^−7^ M acetonitrile solutions of the ligand. The amine guest molecules were in fully protonated forms in water. In the cases of titrations in semi-aqueous media, the HCl salts of the amines were uniformly used, while the acetonitrile solutions of the corresponding neutral amines were added to the solutions of the host compound during the nonaqueous titrations.

**Figure 9:**
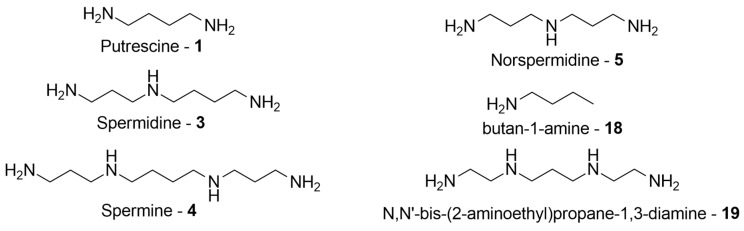
Mono-and oligoamines as investigated guest compounds in spectrofluorimetric molecular recognition studies.

Molecular recognition studies were carried out on selected commercially available mono- and oligoamine derivatives (**1**, **3**, **4**, **5**, **18**, **19**) including the biologically relevant ones. The investigated compounds are shown in Figure 9.

**Figure 10:**
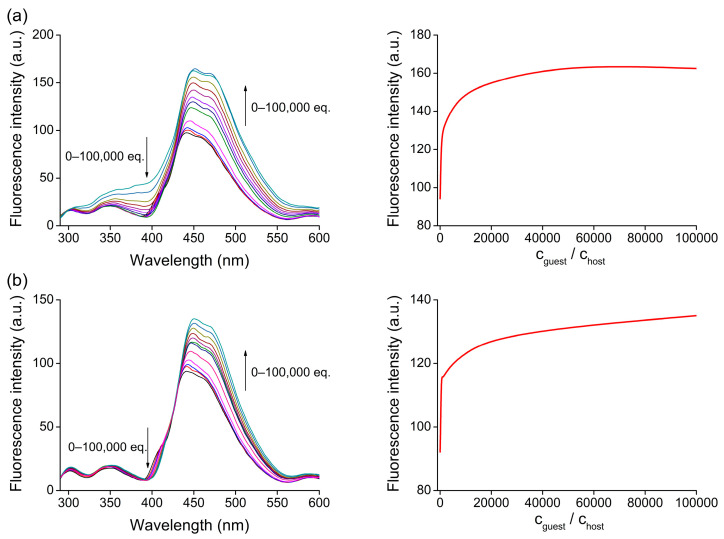
Series of fluorescence emission spectra for titration of **10** with mono- and diamines (**left**) and the corresponding global nonlinear regression curve on fluorescence emission data-series as a function of the concentration of guest molecules (**right**) [(**a**) n-butylamine (**18**); (**b**) putrescine (**1**)].

**Figure 11:**
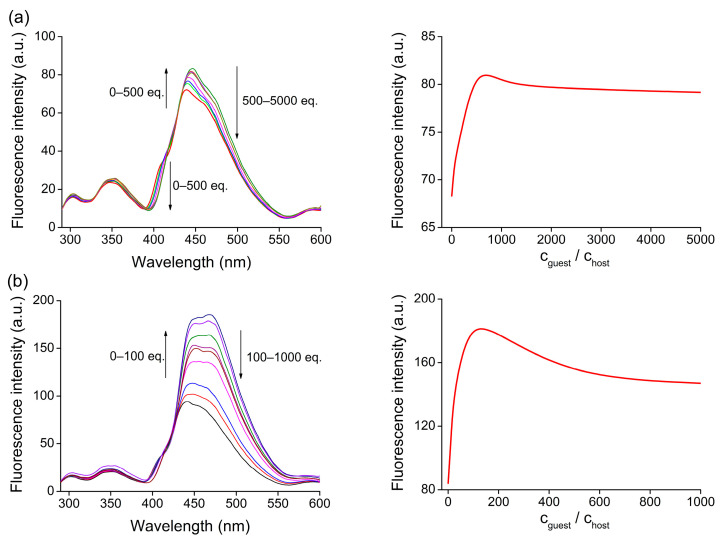
Series of fluorescence emission spectra for titration of **10** with triamines (**left**) and the corresponding global nonlinear regression curve on fluorescence emission data-series as a function of the concentration of guest molecules (**right**) [(**a**) norspermidine (**5**); (**b**) spermidine (**3**)].

**Figure 12:**
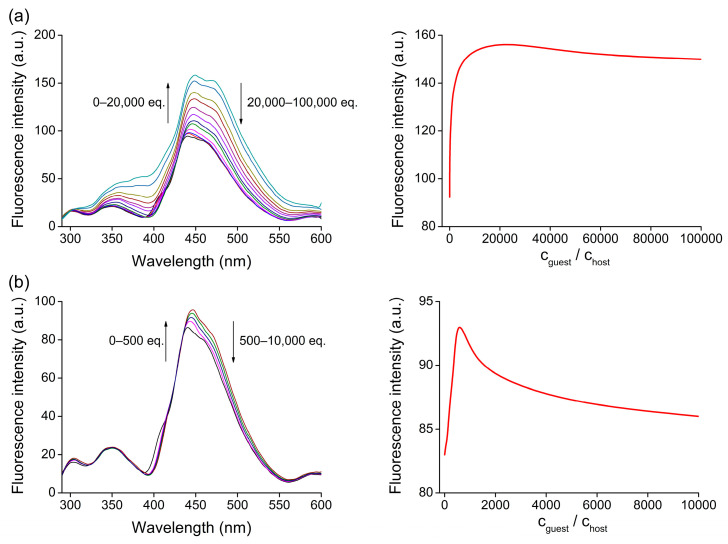
Series of fluorescence emission spectra for titration of **10** with tetraamines (**left**) and the corresponding global nonlinear regression curve on fluorescence emission data-series as a function of the concentration of guest molecules (**right**) [(**a**) *N*,*N*′-bis(2-aminoethyl)-1,3-propanediamine (**19**); (**b**) spermine (**4**)].

Spectrofluorimetric titrations were carried out in a wide range of guest compound concentrations. To determine the structural preference of the host in amine recognition and to calculate the stability constants, global nonlinear regression was applied on the titration datasets. Both the complex stoichiometry and complex stabilities were determined according to the most recent and well-established methods [56,57,58]. The adequacy of the fitted models was checked by statistical *F*-probe. Detailed information regarding the regression methods can be found in the Experimental Section. The results can be seen in Figure 10, Figure 11 and Figure 12.

The results for complex stoichiometry and stability constant calculations based on the regression analyses on titration datasets are summarized in Table 1. The complex stoichiometries are not shown separately in the table, since in all cases the formation of 1:1 and then in a consecutive step 1:2 (host:guest) complexes were observed. This is not surprising as the host molecule contains two macrocycle rings as the main binding sites besides other possibilities to form additional intermolecular non-covalent binding forces. Accordingly, it is obvious that the cooperativity in guest-binding has a considerable effect on the complexation equilibrium.

According to the calculations, the following stability order can be concluded: spermidine~spermine (**4**) > norspermidine (**5**) > *N*,*N*′-bis(2-aminoethyl)-1,3-propanediamine (**19**) > putrescine (**1**) > n-butylamine (**18**). In most cases, cooperativity effects did not take place since there is a homotropic system of the host molecule with two identical binding sites. As the two macrocycle rings of the host molecule are separated from each other, the occupation of the first one practically does not affect the coordination ability of the second one; thus, at most two amine guests can be bonded simultaneously and independently. As the first complexed amine guest can be fixed with additional different non-covalent binding forces within the complex in comparison to the second one, the corresponding log*K*_1_ values are consequently higher. The coordination of the second amine guest starts after the host molecule forms a 1:1 host–guest complex. Thus, it is a stepwise binding process. This complexation behavior is also supported by the observed spectral changes as the 1:2 complexes at the second coordination step result in an opposite-direction in the spectral change. This phenomenon is probably attributed to the termination of the cation-*π* interaction(s) established between the first coordinated ammonium part and the appearance of the more distant acridine unit upon the occupation of the second macrocyclic cavity. However, as the cation-*π* interaction is significantly weaker than the three-podal hydrogen bonding within the macrocycle cavity, the complexation of the first amine guest does not hinder significantly the acceptance of the second one.

The only exception to the binding cooperativity was the case of the spermine-coordination, when regression analysis revealed a negative cooperativity in coordinating the second protonated amine part corresponding to the 1:2 (host:guest) complex stoichiometry. This is attributed to the increased chain length of spermine (**4**) compared to the other oligoamine analogues, which allows the opposite-side terminal protonated primary amino group to be complexed by the second macrocycle ring without a significant structural distortion (see Figure 5). Although the complexation of the first ammonium group hinders the second one to dock into the cavity of the other crown ether ring, it seems to be a slight effect, since the competing ammonium group can push off the previously complexed one from one of the binding sites.

**Figure 13:**
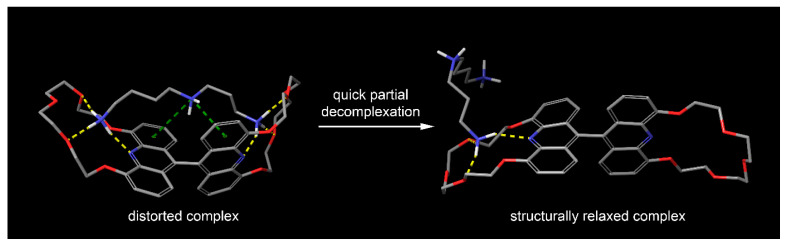
Kinetic instability of 1:2 (host:guest) complexes with shorter-chain amines by the example of coordinating spermidine (**3**).

The latter phenomenon cannot take place regarding the shorter-chain amines, because the double-inclusion complexation in the case of the initial 1:1 stoichiometry causes a conformational distortion of the structure of the host molecule moving the crown ether rings slightly toward each other. This results in a reduced kinetic stability of the complex, which is also supported by the modeling (Figure 13).

Finally, one of the terminal primary ammonium groups departs followed by the conformational relaxation of the host molecule. As revealed by the modeling, the other terminal primary ammonium group remained in the cavity after this partial dissociation of the complex. Thus, the short-lived complexation of both chain ends causes the lack of cooperation effect when forming 1:2 (host:guest) complex with amines of a shorter chain than spermine (**4**).

Formation of complexes with other stoichiometries were not observed. It is not surprising, as more than two protonated amine parts can only be coordinated by cation-*π* interactions with the empty heteroaromatic regions. As the cavity of the 18-crown-6 ether-type ring exactly fits to the size of the primary ammonium ion [43,44], only weaker interactions than the three-podal hydrogen bonding could take place when approaching the guest molecule to the acridine units.

The stability constants for the second-step complexations proved to be smaller than those for the 1:1 complex in accordance with the expectations for a stepwise binding model. In the cases when log*K* values did not reached 2.0, the uncertainty of the fitting process on the titration datasets significantly increased, thus accurate values could not be determined. However, the interactions for these very unstable complexes are too weak to be exploited in any applications.

Some stability constants indicate a very stable complexation among reversible complexing host molecules. Although the high degree of conformational flexibility of aliphatic oligoamines is not favored for complexation from the entropic point of view, the several protonated amine parts and electron-rich acridine units stabilize the complex effectively by increasing the number of coordinative interactions.

The first investigated amine was n-butylamine (**18**), which is the simplest structural subpart of the oligoamines in focus. In this case, only complexation of the primary ammonium group of the guest with the macrocycle ring of the host can take place. According to literature analogues [49], there is a three-podal hydrogen bonding within the complex involving the ammonium protons of the guest and the nucleophilic acridine-*N* atom and two alternating ethereal *O* atoms of the host. It is obvious that only one macroring can take part in the complexation due to the rigid structure of the host. Accordingly, a significantly smaller complex stability constant is expected than those for oligoamines. As we increase the chain length of the oligoamine host, the possibility of further interactions opens up. The protonated secondary amine parts, outside the complexed primary ammonium groups, can establish cation-*π* interactions with one or two acridine units, depending on the chain length of the oligoamine. In parallel, the corresponding complex stabilities are continuously increasing (see Table 1).

Additionally, we also determined the stability constants by titrating with unprotonated amines. It is well known that the lack of the positive charge results in a smaller complex stability constant even if the coordination is possible [43,44], while the additional coordinative interactions with the delocalized *π*-electron system of the acridine units are also missing. The results showed an orders of magnitude decrease in stability constants (see Supplementary Materials).

## 3. Materials and Methods

### 3.1. Chemicals, Preparative Methods, and Characterization of Compounds

Starting materials and reagents were purchased from Sigma-Aldrich Corporation (USA, owned by Merck, Darmstadt, Germany) and used without purification unless otherwise noted. Silica Gel 60 F254 (Merck, Darmstadt, Germany) and aluminum oxide 60 F254 neutral type E (Merck, Germany) plates were used for thin-layer chromatography (TLC). All reactions were monitored by TLC and visualized by UV lamp. Silica Gel 60 (70–230 mesh, Merck) and aluminum oxide 60 F254 neutral type E (Merck, Germany) were used for column chromatography. Purifications by preparative thin-layer chromatography (PTLC) were carried out using silica gel 60 F254 (Merck, Germany) plates of 2 mm layer thickness (art No.: 1.05744) or aluminum oxide 60 F254 neutral type E (Merck, Germany) plates of 0.25 mm layer thickness (art No.: 1.05727). Evaporations were carried out under reduced pressure unless otherwise stated. Ratios of solvents for the eluents are given in volumes (mL/mL).

The new compounds were characterized by their physical constants such as melting point, thin-layer chromatography retention factor (*R*_f_), IR, ^1^H-NMR and ^13^C-NMR spectroscopies, and HRMS spectrometry. Melting points were determined on a Boetius micro-melting point apparatus and are uncorrected. Infrared spectra were recorded on a Bruker Alpha-T FT-IR spectrometer (Bruker Corporation, Billerica, MA, USA). ^1^H- (300 MHz) and ^13^C- (75 MHz) NMR spectra were recorded on a Bruker 300 Avance spectrometer (Bruker Corporation, Billerica, MA, USA). ^1^H- (500 MHz) and ^13^C- (125 MHz) NMR spectra were obtained on a Bruker DRX-500 Avance spectrometer (Bruker Corporation, Billerica, MA, USA). HRMS analysis was carried out on a Laser Ablated Rapid Evaporative Ionization Mass Spectrometry (LA-REIMS) system. LA-REIMS technology employs direct analysis of aerosols generated via laser ablation (OPOTEK OPO laser, λ = 2940 nm, 5 mJ/pulse, 20 Hz, 6 ns pulse width). Then, the aerosol droplets were introduced into the REIMS ion source. Prior to entry into the mass spectrometer, the generated vapor was mixed with propan-2-ol containing leucine-enkephalin, as a lockmass material. The leucine-enkephalin concentration was 0.5 ng/mL and the flow rate was 0.15 mL per min. Raw data were acquired by using a XevoTM G2-XS TOF-MS (Waters Corporation, Wilmslow, UK). The spectra were acquired using positive ion mode. The acquired range was 50–1200 *m*/*z* with 0.5 s/scans. The data were processed with MassLynx V4.2 software.

### 3.2. Molecular Modeling and Structure Optimization

All calculations were carried out with the modules of Schrödinger Suites 2019-2 (Schrödinger, LLC, New York, NY, USA) in Maestro. The 3D structures of the bis(crown ether) and polyamines were generated by LigPrep at pH = 7.4 using OPLS3e force field. Polyamines were manually docked to the bis(crown ether) in fully protonated forms. The complexes were minimized, and water boxes were constructed for molecular dynamics simulations. Net positive charge of the ligands was neutralized by chloride ions. SPC solvent model was used. Boundaries were adjusted to 10 Å from the complexes in each axis, resulting in an orthorhombic box. The systems were relaxed before simulation using the default relaxation process of Desmond. 

Complexes obtained from the first frame of the MD simulations were selected for structure optimization. Water molecules and chloride ions were removed. Structure optimization of the complexes was conducted by density functional theory (DFT), using the B3LYP-D3 functional. Optimizations were carried out using fully analytical accuracy. The calculations were carried out using PBF solvent model and water as solvent.

### 3.3. Spectroscopic Studies on Molecular Recognition

UV–Vis spectra were recorded on a UNICAM UV4-100 spectrophotometer controlled by VIZION 3.4 software (ATI UNICAM, Knutsford, UK). Fluorescence emission spectra were recorded on a Perkin-Elmer LS 50B luminescent spectrometer (PerkinElmer Inc., USA) and were corrected by FL Winlab 3.0 spectrometer software (PerkinElmer Inc., Shelton, CT, USA). Quartz cuvettes with a path length of 1 cm were used in all cases. Spectroscopic measurements were carried out at room temperature (25  ±  1 °C). Polarizers were not applied. A 290 nm cut-off type bandpass filter and 10 nm excitation and emission slits were used in the cases of titration experiments. The solutions were added with a Hamilton-syringe to the 10^−7^ M acetonitrile solutions of the ligand. The results were corrected with the background signal and the dilution effect of the added solutions. The analyte solution was added until constant optical signal was observed, which state was defined as the end point of the titration experiment. In the case of fluorescence measurements, an excitation wavelength of 265 nm was applied corresponding to the absorption peak-wavelength of the host molecule (absorption spectrum of the host molecule can be found in the Supplementary Materials).

The stability constants of the complexes (*K*) were determined by global non-linear regression analysis. For determination of the complex stability constant based on the observed fluorescence enhancement upon complexation, the following equation was used [56]:

(1) $$F=I_{0}\Phi \varepsilon b\left[X\right]=k_{X}\left[X\right],$$

where *F* is the measured fluorescence intensity, *I*_0_ is the intensity of the emission, *Φ* is the fluorescence quantum yield, *ε* is the molar absorption coefficient, *b* is the optical path length, [*X*] is the molar concentration of species *X*, and *k_X_* is a constant referring to the optical properties of species *X*.

In the case of complexes with 1:1 stoichiometry, the association constant can be calculated by the following equation [56]:

(2) $$\frac{F}{F_{0}}=\frac{k_{H}/k_{H}^{0}+\left(k_{HG}/k_{H}^{0}\right)K_{a}\left[G\right]}{1+K_{a}[G]},$$

where the ratios of *k* parameters and *K* were left as floating parameters during the fitting method. Parameters *F* and *F*_0_ are wavelength-dependent variables and [*G*] was set as a variable, too. *F*_0_ refers to the initial fluorescence intensity of the free host molecule, *k_H_* is a constant referring to the optical properties of the free host molecule, $k_{H}^{0}$ is a constant referring to the optical properties of the free host in the presence of preferred guest molecules, constant *k_HG_* describes the photophysical features of the complex, *K* is the association constant, and [*G*] is the concentration of the initial guest molecules.

Global non-linear fitting was carried out similarly in the case of complexes with 1:2 (host:guest) stoichiometry based on the following equation [56]:

(3) $$∆F=\frac{k_{∆HG}{\left[H\right]}_{0}K_{1}\left[G\right]+k_{∆HG_{2}}{\left[H\right]}_{0}K_{1}K_{2}{\left[G\right]}^{2}}{1+K_{1}\left[G\right]+K_{1}K_{2}{\left[G\right]}^{2}}$$

where Δ*F* is the change in fluorescence during titration steps, *k*_Δ*HG*_ = *k_HG_* − *k_H_*, [*H*]_0_ is the initial concentration of the host, *K*_1_ is the stability constant for the first step, while *K*_2_ is that for the second step of the complexation.

For determinations of the complex stoichiometries were also carried out by applying the described global non-linear fitting methods. These results were compared in terms of the quality of fit indicators according to recent literature suggestions [57,58]. The choice of the model was made by a statistical *F*-probe, which aimed to test the sum of squares from each model fitting. In the cases of formulas for 1:1 and 1:2 (host:guest) stoichiometries, the observed *F*-values supported the null hypothesis where the fitted model described an appropriate relationship for characterizing the population of experimental data at a confidence level of 95%. In contrast, in the cases of models for other stoichiometries, the test showed that the applied models did not fit the experimental data.

As a composite binding model, including a 1:1 and 1:2 (host:guest) stepwise coordinations, was obtained in all of the cases, the two sections of the titration curves with an opposite direction fluorescence response (turn-on fluorescence response and a subsequent partial quenching) can be well fitted with the linear combinations of the following two mathematical formulas (weighted sum of Equations (2) and (3) with some rearrangements [56]):

(4) $$F={c(F}_{0}\frac{k_{H}/k_{H}^{0}+\left(k_{HG}/k_{H}^{0}\right)K_{a}\left[G\right]}{1+K_{a}[G]})+\left(1-c\right)[F_{0}-\left(F_{0}-F_{c}\right)\frac{{\left[H\right]}_{0}+\left[G\right]+\frac{1}{K_{a}}-\sqrt{{\left({\left[H\right]}_{0}+\left[G\right]+\frac{1}{K_{a}}\right)}^{2}-4{\left[H\right]}_{0}\left[G\right]}}{2{\left[H\right]}_{0}}],$$

where *c* is the linear coefficient, which varies as a function of [G], while *F_c_* is the fluorescence of the fully complexed host.

OriginPro 8.6 (OriginLab Corp., Northampton, MA, USA) software was used for evaluating the spectrophotometric measurements. 

### 3.4. Synthesis of Compounds

#### 3.4.1. Synthesis and Optimized Workup for 9-Chloro-4,5-dimethoxyacridine (**12**)

4,5-dimethoxyacridone (**11**) (1.50 g, 5.88 mmol) and phosphorus pentachloride (3.68 g, 17.65 mmol) were mixed in phosphoryl chloride (8 mL, 88.20 mmol) and the temperature of the resulting mixture was raised to 90 °C and stirred at this value for 4 h under an argon atmosphere. The volatile components were removed, and the residue was taken up in dichloromethane (100 mL). The latter solution was slowly poured into a vigorously stirred 40 m/m% aqueous trimethylamine solution (50 mL), with an external cooling of salt and ice to keep the internal temperature of the reaction mixture below 5 °C. After destroying the excess reagent, the phases were separated, and the aqueous phase was extracted with dichloromethane (5 × 50 mL). The combined organic phase was dried over magnesium sulfate, filtered, and the solvent was removed. The crude product was purified by column chromatography on aluminum oxide using dichloromethane/hexane 1:1 as eluent. The yellow crystalline product was obtained almost quantitatively (1.58 g; 98%) 

Rf = 0.48 (silica gel TLC, ethyl acetate). The other data for the characterization of 9-chloro-4,5-dimethoxyacridine concurred with those reported [52,53].

#### 3.4.2. 4,4′,5,5′-Tetramethoxy-9,9′-biacridine (**14**)

A vigorously stirred suspension of chloroacridine **12** (1.98 g, 7.24 mmol) in dry and pure tetrahydrofuran (40 mL) was cooled down to −10 °C under an argon atmosphere, and then palladium acetate (113.8 mg, 0.51 mmol, 0.07 equiv.) was added to it. Freshly made phenylmagnesium bromide (2 M in diethyl ether, 6.00 equiv.) and dilithium tetrachlorocuprate (0.1 M in THF, 0.01 equiv.) were added dropwise to the stirred mixture, while keeping the temperature between 0 and −10 °C. After addition, the reaction mixture was allowed to warm up to room temperature, and then its temperature was raised to 60 °C and kept at this value for 20 h. After this time, the reaction mixture was cooled down to 20 °C. The volatile components were removed, and the residue was then taken up in ethyl acetate (50 mL) and saturated aqueous ammonium chloride solution (50 mL). The phases were shaken well and separated. The solid precipitate was filtered off from the organic phase and washed with ethyl acetate. The ethyl acetate phase and washings were dried over anhydrous magnesium sulfate, filtered, and evaporated. The crude product was purified by column chromatography on silica gel using methanol/ethyl acetate 1:100 as eluent. The pure product was obtained as yellowish-brown crystals (1.22 g; 71%).

m.p. = Decomposition before melting (336 °C) R_f_ = 0.7 (silica gel TLC, dichloromethane/methanol 30:1). ^1^H-NMR (500 MHz, CDCl_3_): δ [ppm]: 7.22 (t, *J* = 10 Hz, 4H), 7.02 (d, *J* = 10 Hz, 4H), 6.60 (d *J* = 10 Hz, 4H), 4.20 (s, 12H). ^13^C-NMR (125 MHz, CDCl_3_): δ [ppm]: 155.8, 140.7, 127.1, 126.8, 117.6, 106.1, 100.0, 56.2. IR: ν_max_ [cm^−1^]: 3388, 3117, 3016, 2805, 1749, 1636, 1565, 1438, 1398, 1319, 1272, 1204, 1137, 1083, 976, 744, 716, 634, 609 HR-MS: *m*/*z* = [M + H]^+^: 477.1822 (Calcd. for C_30_H_24_N_2_O_4_, 477.1814).

#### 3.4.3. [9,9′-Biacridine]-4,4′,5,5′-tetraol (**15**)

To 4,4′,5,5′-tetramethoxy-9,9′-biacridine (**14**) (500 mg, 1.05 mmol) pyridinium chloride (7 g, 60.58 mmol) was added, and the temperature of this mixture was raised to 220 °C. The reaction mixture was stirred at this temperature for 2 h, and then it was poured into cold distilled water (150 mL) and stirred for an additional 1 h. The precipitate was filtered, washed with cold distilled water (3 × 15 mL), and it was taken up in a mixture of ethyl acetate (50 mL) and saturated aqueous sodium acetate solution (50 mL). The phases were shaken well and separated. The aqueous phase was extracted with ethyl acetate (3 × 50 mL). The combined organic phase was dried over magnesium sulfate, filtered, and the solvent was removed. The crude product was purified by recrystallization from a propan-2-ol/diethyl ether mixture. The pure product was obtained as dark yellow crystals (331 mg; 75%).

m.p. ≥ 365 °C R_f_ = 0.24 (silica gel TLC, dichloromethane/methanol 30:1). ^1^H-NMR (500 MHz, DMSO-*d*_6_): δ [ppm]: 10.51 (s, 4H), 7.23 (t, *J* = 10 Hz, 4H), 7.12 (d, *J* = 10 Hz, 4H), 6.43 (d, *J* = 10 Hz, 4H). ^13^C-NMR (125 MHz, DMSO-*d*_6_): δ [ppm]: 151.6, 141.9, 138.1, 128.1, 126.5, 116.9, 109.8. IR: ν_max_ [cm^−1^]: 3351, 2923, 2853, 1703, 1664, 1574, 1463, 1376, 1334, 1260, 1211, 1175, 1096, 1041, 901, 884, 843, 800, 748, 719, 666, 535, 483. HR-MS: *m*/*z* = [M + H]^+^: 421.1198 (Calcd. for C_26_H_16_N_2_O_4_, 421.1188)

#### 3.4.4. 27-Chloro-6,9,12,15,18-pentaoxa-25-azatetracyclo[21.3.1.0^5,26^.0^19,24^]heptacosa 1,3,5(26),19,21,23(27),24-heptaene (**17**)

Acridono-crown ether **16** (101 mg, 0.26 mmol) and phosphorus pentachloride (163 mg, 0.79 mmol) were mixed in phosphoryl chloride (1.2 mL, 13.23 mmol). The temperature of this mixture was raised to 90 °C and stirred at this value for 4 h under an argon atmosphere. After the reaction was completed, the volatile components were removed, and the residue was taken up in dichloromethane (100 mL). This solution was slowly poured into a vigorously stirred 40 m/m% aqueous trimethylamine solution (20 mL) with an external cooling of salt and ice to keep the internal temperature of the mixture below 5 °C. After destroying the excess reagent, the phases were separated, and the aqueous phase was extracted with dichloromethane (5 × 20 mL). The combined organic phase was dried over anhydrous magnesium sulfate, filtered, and the solvent was removed. The crude product was purified by column chromatography on aluminum oxide using dichloromethane as eluent. The yellow crystalline product was obtained almost quantitatively (102 mg; 97%)

m.p. = 139–141 °C. R_f_ = 0.21 (silica gel TLC, dichloromethane/methanol 30:1). ^1^H-NMR (500 MHz, CDCl_3_): δ [ppm]: 7.97 (d, *J* = 10 Hz, 2H), 7.53 (t, *J* = 10 Hz, 2H), 7.01 (d, *J* = 10 Hz, 2H), 4.41 (t, *J* = 5 Hz, 4H), 4.19 (t, *J* = 5 Hz, 4H), 3.97–3.95 (m, 4H), 3.87–3.85 (m, 4H). ^13^C-NMR (125 MHz, CDCl_3_): δ [ppm]: 155.2, 141.0, 140.2, 127.4, 125.7, 116.2, 107.4, 72.1, 70.5, 69.4, 69.0. IR: ν_max_ [cm^−1^]: 2922, 2853, 1620, 1557, 1447, 1417, 1393, 1362, 1326, 1276, 1260, 1211, 1192, 1113, 1082, 1043, 909, 862, 848, 814, 744, 773, 690, 646, 574, 509.

#### 3.4.5. 27-{6,9,12,15,18-Pentaoxa-25-azatetracyclo[21.3.1.0^5,26^.0^19,24^]heptacosa-1(27),2,4,19,21,23,25-heptaen-27-yl}-6,9,12,15,18-pentaoxa-25-azatetracyclo[21.3.1.0^5,26^.0^19,24^]heptacosa-1,3,5(26),19,21,23(27),24-heptaene (**10**)

##### Preaparation from [9,9′-Biacridine]-4,4′,5,5′-tetraol (**15**)

To biacridine-tetraol **15** (160 mg, 0.38 mmol), ethylene glycol ditosylate (924 mg, 1.80 mmol), potassium carbonate (535 mg, 3.80 mmol), and DMF (16 mL) were added under argon atmosphere, and the temperature of this vigorously stirred suspension was raised to 80 °C. The reaction mixture was stirred at this temperature for 7 days. The solvent was removed, and the residue was taken up in distilled water (10 mL) and dichloromethane (25 mL). The phases were shaken well and separated. The aqueous phase was extracted with dichloromethane (3 × 10 mL). The combined organic phase was dried over magnesium sulfate, filtered, and the solvent was removed. The crude product was first purified by column chromatography on aluminum oxide using toluene/ethanol 5:2 mixture as eluent then by PTLC on aluminum oxide using the same eluent to give bis(crown ether) **10** as bright yellow crystals. (16.80 mg; 6%)

m.p. = 103–106 °C. R_f_ = 0.7 (aluminum oxide TLC, toluene/ethanol 1:1). ^1^H-NMR (500 MHz, CDCl_3_): δ [ppm]: 7.97 (d, *J* = 10 Hz, 4H), 7.53 (t, *J* = 10 Hz, 4H), 7.03 (d, *J* = 10 Hz, 4H), 4.44 (t, *J* = 5 Hz, 8H), 4.20 (t, *J* = 5 Hz, 8H), 3.92–3.90 (m, 8H), 3.90–3.80 (m, 8H). ^13^C-NMR (125 MHz, CDCl_3_): δ [ppm]: 154.4, 140.8, 140.7, 127.3, 125.7, 116.5, 107.6, 71.0, 70.2, 68.6, 68.5. IR: ν_max_ [cm^−1^]: 3400, 2922, 2853, 1722, 1623, 1558, 1532, 1471, 1453, 1417, 1395, 1364, 1327, 1274, 1258, 1197, 1093, 1042, 917, 853, 813, 741, 568. HRMS: *m*/*z* = [M + H]^+^: 737.3090 (Calcd. for C_42_H_44_N_2_O_10_, 737.3074)

##### Preaparation from 27-Chloro-6,9,12,15,18-pentaoxa-25-azatetracyclo[21.3.1.0^5,26^.0^19,24^]heptacosa 1,3,5(26),19,21,23(27),24-heptaene (**17**)

Freshly prepared chloroacridino-crown ether **17** (213 mg, 0.57 mmol) was vigorously stirred in *tert-*butylbenzene (4.26 mL) under argon atmosphere. Zinc powder (44.85 mg, 0.69 mmol) was added to this mixture and the temperature of it was raised to 90 °C, and the stirring continued at this value for 15 h. After the reaction was completed, the solvent was removed, and the residue was taken up in distilled water (20 mL) and dichloromethane (20 mL). The phases were shaken well and separated. The aqueous phase was extracted with dichloromethane (3 × 20 mL). The combined organic phase was dried over magnesium sulfate, filtered, and the solvent was removed. The crude product was first purified by column chromatography on aluminum oxide using toluene/ethanol 5:2 mixture as eluent, and then by PTLC on aluminum oxide using the same eluent to give bis(crown ether) **10** as bright yellow crystals. (14.7 mg; 7%). Bis(crown-ether) **10** prepared in this way was identical compared to that obtained by the previous procedure.

The only byproduct was acridono-crown ether **16**, which was formed by the hydro-lysis of the starting material chloroacridino-crown ether **17**, and it was purified by recrystallization from *tert*-butylbenzene.

## 4. Conclusions

The DFT-based modeling predicted well the experimentally determined complex stabilities. The preference order of the host molecule in molecular recognition was as follows: spermine (**4**)~spermidine (**3**) > norspermidine (**5**) > *N*,*N*′-bis(2-aminoethyl)-1,3-propanediamine (**19**) > putrescine (**1**) > n-butylamine (**18**). The difference in stability between spermine (**4**) and spermidine (**3**) complexes was not found to be significant. Overall, the coordination of spermine (**4**) is considered the most favorable one, since in this case the structural distortion of the host molecule is the smallest compared to its uncomplexed conformation. Moreover, the most independent attractive interactions form here, and negative cooperativity also takes place in the composite binding model. Three *C*-*C* bonds between the terminal primary ammonium groups and the interchain protonated amine parts, while four *C*-*C* bonds between the two interchain protonated amine parts represent the optimal distances in terms of creating intermolecular interactions. Amino groups with smaller distances reduce the number of coordination possibilities, since the structure of the host molecule is quite rigid. In practice, a difference of at least one order of magnitude between the complex stability constants is typically required in order to achieve effective molecular discrimination in separation or sensing. Accordingly, tri- and tetraamine-type spermine analogues can be selectively recognized in the presence of di- and mono-type ones. The host molecule reported here is a promising one for the studies of oligoamine-centered processes, which are among the focal points of modern biochemical research.

## Figures and Tables

**Table 1:** Calculated stability constants and cooperativity in binding for the determined consecutive 1:1 and 1:2 (host:guest) amine-coordination of the host compound.

Amine Guest ^1^	log*K*_1_ ^2^	log*K*_2_ ^3^	Binding Cooperativity Effects ^4^
n-butylamine (**18**)	2.1 ± 0.2	<<2.0	non-cooperative
Putrescine (**1**)	2.6 ± 0.2	<<2.0	non-cooperative
*N*,*N*′-bis(2-aminoethyl) -1,3-propanediamine (**19**)	3.7 ± 0.2	3.0 ± 0.2	non-cooperative
Norspermidine (**5**)	5.1 ± 0.1	4.5 ± 0.1	non-cooperative
Spermine (**4**)	5.3 ± 0.1	4.4 ± 0.1	negative cooperativity (*α* = 0.6)
Spermidine (**3**)	5.5 ± 0.1	4.8 ± 0.1	non-cooperative

^1^ Added in aqueous solution in a form of HCl salt (fully protonated with a Cl^−^ counterion for all of the amino groups). ^2^ Complex stability constant for 1:1 (host:guest) complexes as the first step of coordination. ^3^ Complex stability constant for 1:2 (host:guest) complexes as the second step of coordination. ^4^ Calculated according to [56], where *K*_1_ = 4∙*K*_2_ refers to a non-cooperative stepwise binding, while *α* expresses the extent of cooperativity.

## Data Availability

The authors confirm that the data supporting the findings of this study are available within the article and its Supplementary Materials.

## Supplementary Materials

The following supporting information can be downloaded at: https://www.mdpi.com/article/10.3390/molecules29184390/s1, Figure S1: ^1^H-NMR spectrum of **14** (CDCl_3_); Figure S2: ^13^C-NMR spectrum of **14** (CDCl_3_); Figure S3: ^1^H-NMR spectrum of **15** (DMSO-*d*_6_); Figure S4: ^13^C-NMR spectrum of **15** (DMSO-*d*_6_); Figure S5: ^1^H-NMR spectrum of **17** (CDCl_3_); Figure S6: ^13^C-NMR spectrum of **17** (CDCl_3_); Figure S7: ^1^H-NMR spectrum of **10** (CDCl_3_); Figure S8: ^13^C-NMR spectrum of **10** (CDCl_3_); Figure S9: UV spectrum of **10** in acetonitrile; Table S1: Calculated stability constants and cooperativity in binding for the determined consecutive 1:1 and 1:2 (host:guest) amine-coordination of the host molecule.

## References

[1] Santos M.H.S. (1996). Biogenic Amines: Their Importance in Foods. Int. J. Food Microbiol..

[2] Givanoudi S., Heyndrickx M., Depuydt T., Khorshid M., Robbens J., Wagner P. (2023). A Review on Bio- and Chemosensors for the Detection of Biogenic Amines in Food Safety Applications: The Status in 2022. Sensors.

[3] Davídek J. (2018). Natural Toxic Compounds of Foods.

[4] Schuber F. (1989). Influence of Polyamines on Membrane Functions. Biochem. J..

[5] Agostinelli E., Arancia G., Vedova L.D., Belli F., Marra M., Salvi M., Toninello A. (2004). The Biological Functions of Polyamine Oxidation Products by Amine Oxidases: Perspectives of Clinical Applications. Amino Acids.

[6] Ma W., Chen K., Li Y., Hao N., Wang X., Ouyang P. (2017). Advances in Cadaverine Bacterial Production and Its Applications. Engineering.

[7] Pegg A.E. (2014). The Function of Spermine. IUBMB Life.

[8] Pegg A.E. (2016). Functions of Polyamines in Mammals. J. Biol. Chem..

[9] Peng Q., Wong C.Y.-P., Cheuk I.W., Teoh J.Y.-C., Chiu P.K.-F., Ng C.-F. (2021). The Emerging Clinical Role of Spermine in Prostate Cancer. Int. J. Mol. Sci..

[10] Proctor M.S., Fletcher H.V., Shukla J.B., Rennert O.M. (1975). Elevated Spermidine And Spermine Levels In The Blood Of Psoriasis Patients. J. Investig. Dermatol..

[11] Saiki S., Sasazawa Y., Fujimaki M., Kamagata K., Kaga N., Taka H., Li Y., Souma S., Hatano T., Imamichi Y. (2019). A Metabolic Profile of Polyamines in Parkinson Disease: A Promising Biomarker. Ann. Neurol..

[12] Han W., Li H., Chen B. (2022). Research Progress and Potential Applications of Spermidine in Ocular Diseases. Pharmaceutics.

[13] Thomas T., Thomas T.J. (2003). Polyamine Metabolism and Cancer. J. Cell. Mol. Med..

[14] Palmer A.J., Wallace H.M. (2010). The Polyamine Transport System as a Target for Anticancer Drug Development. Amino Acids.

[15] Vanhoutte R., Kahler J.P., Martin S., van Veen S., Verhelst S.H.L. (2018). Clickable Polyamine Derivatives as Chemical Probes for the Polyamine Transport System. ChemBioChem.

[16] Ni Y.-Q., Liu Y.-S. (2021). New Insights into the Roles and Mechanisms of Spermidine in Aging and Age-Related Diseases. Aging Dis..

[17] Al-Habsi M., Chamoto K., Matsumoto K., Nomura N., Zhang B., Sugiura Y., Sonomura K., Maharani A., Nakajima Y., Wu Y. (2022). Spermidine Activates Mitochondrial Trifunctional Protein and Improves Antitumor Immunity in Mice. Science.

[18] Tao X., Liu J., Diaz-Perez Z., Foley J.R., Nwafor A., Stewart T.M., Casero R.A., Zhai R.G. (2024). Reduction of Spermine Synthase Enhances Autophagy to Suppress Tau Accumulation. Cell Death Dis..

[19] Tiburcio A.F., Alcázar R. (2018). Potential Applications of Polyamines in Agriculture and Plant Biotechnology. Methods Mol. Biol..

[20] Issaq H.J. (1999). Capillary Electrophoresis of Natural Products-II. Electrophoresis.

[21] Oguri S. (2000). Electromigration Methods for Amino Acids, Biogenic Amines and Aromatic Amines. J. Chromatogr. B Biomed. Sci. Appl..

[22] Chiu T., Lin Y., Huang Y., Chang H. (2006). Analysis of Biologically Active Amines by CE. Electrophoresis.

[23] Zhang Y., Zhang Y., Zhou Y., Li G., Yang W., Feng X. (2019). A Review of Pretreatment and Analytical Methods of Biogenic Amines in Food and Biological Samples since 2010. J. Chromatogr. A.

[24] Chiu P.K.-F., Fung Y.-H., Teoh J.Y.-C., Chan C.-H., Lo K.-L., Li K.-M., Tse R.T.-H., Leung C.-H., Wong Y.-P., Roobol M.J. (2021). Urine Spermine and Multivariable Spermine Risk Score Predict High-Grade Prostate Cancer. Prostate Cancer Prostatic Dis..

[25] Krämer J., Kang R., Grimm L.M., De Cola L., Picchetti P., Biedermann F. (2022). Molecular Probes, Chemosensors, and Nanosensors for Optical Detection of Biorelevant Molecules and Ions in Aqueous Media and Biofluids. Chem. Rev..

[26] He X., Ding F., Sun X., Zheng Y., Xu W., Ye L., Chen H., Shen J. (2021). Reversible Chemosensor for Bioimaging and Biosensing of Zn(II) and HpH in Cells, Larval Zebrafish, and Plants with Dual-Channel Fluorescence Signals. Inorg. Chem..

[27] Martínez-Máñez R., Sancenón F. (2003). Fluorogenic and Chromogenic Chemosensors and Reagents for Anions. Chem. Rev..

[28] Martínez-Máñez R., Sancenón F. (2006). Chemodosimeters and 3D Inorganic Functionalised Hosts for the Fluoro-Chromogenic Sensing of Anions. Coord. Chem. Rev..

[29] Wu D., Sedgwick A.C., Gunnlaugsson T., Akkaya E.U., Yoon J., James T.D. (2017). Fluorescent Chemosensors: The Past, Present and Future. Chem. Soc. Rev..

[30] Tanima D., Imamura Y., Kawabata T., Tsubaki K. (2009). Development of Highly Sensitive and Selective Molecules for Detection of Spermidine and Spermine. Org. Biomol. Chem..

[31] Lee B., Scopelliti R., Severin K. (2011). A Molecular Probe for the Optical Detection of Biogenic Amines. Chem. Commun..

[32] Nakamura M., Sanji T., Tanaka M. (2011). Fluorometric Sensing of Biogenic Amines with Aggregation-Induced Emission-Active Tetraphenylethenes. Chem. Eur. J..

[33] Barros M., Ceballos S., Arroyo P., Sáez J.A., Parra M., Gil S., Costero A.M., Gaviña P. (2021). Spermine and Spermidine Detection through Restricted Intramolecular Rotations in a Tetraphenylethylene Derivative. Chemosensors.

[34] Köstereli Z., Severin K. (2012). Fluorescence Sensing of Spermine with a Frustrated Amphiphile. Chem. Commun..

[35] Satrijo A., Swager T.M. (2007). Anthryl-Doped Conjugated Polyelectrolytes as Aggregation-Based Sensors for Nonquenching Multicationic Analytes. J. Am. Chem. Soc..

[36] Chow C.-F., Lam M.H.W., Wong W.-Y. (2013). Design and Synthesis of Heterobimetallic Ru(II)–Ln(III) Complexes as Chemodosimetric Ensembles for the Detection of Biogenic Amine Odorants. Anal. Chem..

[37] Khan S.A., Misra T.K. (2022). Novel Gluconate Stabilized Gold Nanoparticles as a Colorimetric Sensor for Quantitative Evaluation of Spermine. Eng. Asp..

[38] Rawat K.A., Bhamore J.R., Singhal R.K., Kailasa S.K. (2017). Microwave Assisted Synthesis of Tyrosine Protected Gold Nanoparticles for Dual (Colorimetric and Fluorimetric) Detection of Spermine and Spermidine in Biological Samples. Biosens. Bioelectron..

[39] Fletcher J.T., Bruck B.S. (2015). Spermine Detection via Metal-Mediated Ethynylarene ‘Turn-on’ Fluorescence Signaling. Sens. Actuators. B Chem..

[40] Lu B., Wang L., Ran X., Tang H., Cao D. (2022). Recent Advances in Fluorescent Methods for Polyamine Detection and the Polyamine Suppressing Strategy in Tumor Treatment. Biosensors.

[41] Maynor M.S., Nelson T.L., O’Sulliva C., Lavigne J.J. (2007). A Food Freshness Sensor Using the Multistate Response from Analyte-Induced Aggregation of a Cross-Reactive Poly (Thiophene). Org. Lett..

[42] Singh P., Mittal L.S., Bhargava G., Kumar S. (2017). Ionic Self-Assembled Platform of Perylenediimide–Sodium Dodecylsulfate for Detection of Spermine in Clinical Samples. Chem. Asian J..

[43] Buschmann H.-J., Schollmeyer E., Mutihac L. (1998). The Complexation of the Ammonium Ion by 18-Crown-6 in Different Solvents and by Noncyclic Ligands, Crown Ethers and Cryptands in Methanol. Supramol. Sci..

[44] Buschmann H.-J., Mutihac L., Mutihac R. (1999). Physicochemical Parameters of the Transport of Amines and Amino Acids through Liquid Membranes by Macrocyclic Ligands. Sep. Sci. Technol..

[45] Newcomb M., Timko J.M., Walba D.M., Cram D.J. (1977). Host-Guest Complexation. 3. Organization of Pyridyl Binding Sites. J. Am. Chem. Soc..

[46] Saaid M., Saad B., Rahman I.A., Ali A.S.M., Saleh M.I. (2010). Extraction of Biogenic Amines Using Sorbent Materials Containing Immobilized Crown Ethers. Talanta.

[47] Udvarhelyi P.M., Sunter D.C., Watkins J.C. (1990). Direct Separation of Amino Acid Enantiomers Using a Chiral Crown Ether Stationary Phase. J. Chromatogr. A.

[48] Kovács E., Deme J., Turczel G., Nagy T., Farkas V., Trif L., Kéki S., Huszthy P., Tuba R. (2019). Synthesis and Supramolecular Assembly of Fluorinated Biogenic Amine Recognition Host Polymers. Polym. Chem..

[49] Vezse P., Benda B., Fekete A., Golcs Á., Tóth T., Huszthy P. (2022). Covalently Immobilizable Tris(Pyridino)-Crown Ether for Separation of Amines Based on Their Degree of Substitution. Molecules.

[50] Mao L., Liu Y., Yang S., Li Y., Zhang X., Wei Y. (2019). Recent Advances and Progress of Fluorescent Bio-/Chemosensors Based on Aggregation-Induced Emission Molecules. Dye. Pigment..

[51] Huszthy P., Köntös Z., Vermes B., Pintér Á. (2001). Synthesis of Novel Fluorescent Acridono- and Thioacridono-18-Crown-6 Ligands. Tetrahedron.

[52] Golcs Á., Ádám B.Á., Vezse P., Huszthy P., Tóth T. (2021). Synthesis and Spectrophotometric Studies of 9-Substituted-4,5-dimethoxyacridine Multifunctionalizable Fluorescent Dyes and Their Macrocyclic Derivatives. Eur. J. Org. Chem..

[53] Németh T., Tóth T., Balogh G.T., Huszthy P. (2017). Synthesis and Fluorescence Spectroscopic Studies of Novel 9-phenylacridino-18-crown-6 Ether Type Sensor Molecules. Period. Polytech. Chem. Eng..

[54] Huszthy P., Vermes B., Báthori N., Czugler M. (2003). Synthesis and X-Ray Crystallographic Studies of Novel Proton-Ionizable Nitro- and Halogen-Substituted Acridono-18-Crown-6 Chromo- and Fluorogenic Ionophores. Tetrahedron.

[55] Boyer G., Lormier T., Galy J.-P., Llamas-Saiz A.L., Foces- Foces C., Fierros M., Elguero J., Virgili A. (1999). X-ray Crystallography at 170 K of Racemic 2,2′-Dimethoxy-9,9′-biacridine and 1H NMR Study of 2,2′-Diacetoxy-9,9′-biacridine. Molecules.

[56] Thordarson P. (2011). Determining association constants from titration experiments in supramolecular chemistry. Chem. Soc. Rev..

[57] Van de Weert M., Stella L. (2011). Fluorescence quenching and ligand binding: A critical discussion of a popular methodology. J. Mol. Struct..

[58] Ulatowski F., Dabrowa K., Bałakier T., Jurczak J. (2016). Recognizing the limited applicability of Job plots in studying host–guest interactions in supramolecular chemistry. J. Org. Chem..

