# Supplementary material for: A Bis(Acridino)-Crown Ether for Recognizing Oligoamines in Spermine Biosynthesis
Source: Molecules. 2024 Sep 15;29(18):4390. doi: 10.3390/molecules29184390 (PMC11433836; doi:10.3390/molecules29184390)
Supplement: Supplementary file 1 [file molecules-29-04390-s001.zip › molecules-3164909-supplementary.pdf]

Supplementary Material

A Bis(Acridino)-Crown Ether for Recognizing Oligoamines in Spermine Biosynthesis

Péter Kisfaludi [1], Sára Spátay [1,2], Marcell Krekó [2,3], Panna Vezse [1], Tünde Tóth [1,4], Péter Huszthy [1,*] and Ádám Golcs [1,2,3,*]

1. Department of Organic Chemistry and Technology, Budapest University of Technology and Economics, Szent Gellért Square 4, 1111 Budapest, Hungary
2. Department of Pharmaceutical Chemistry, Semmelweis University, Hőgyes Endre Street 9, 1092 Budapest, Hungary
3. Center for Pharmacology and Drug Research & Development, Department of Pharmaceutical Chemistry, Semmelweis University, Üllői Street 26, 1085 Budapest, Hungary
4. HUN-REN Centre for Energy Research, Konkoly-Thege Miklós Street 29-33, 1121 Budapest, Hungary

* Correspondence: huszthy.peter@vbk.bme.hu (P.H.); golcs.adam@semmelweis.hu (Á.G.)

Contents

1. ^{1}H-NMR and ^{13}C-NMR spectra of the new compounds and the previously reported, but not characterized compound **17**

2. UV-spectroscopic investigation of the new crown ether (**10**)

3. Results for neutral oligoamines

4. Reported data for the characterization of 9-chloro-4,5-dimethoxyacridine [52,53]

1. ^{1}H-NMR and ^{13}C-NMR spectra of the new compounds and the previously reported, but not characterized compound **17**

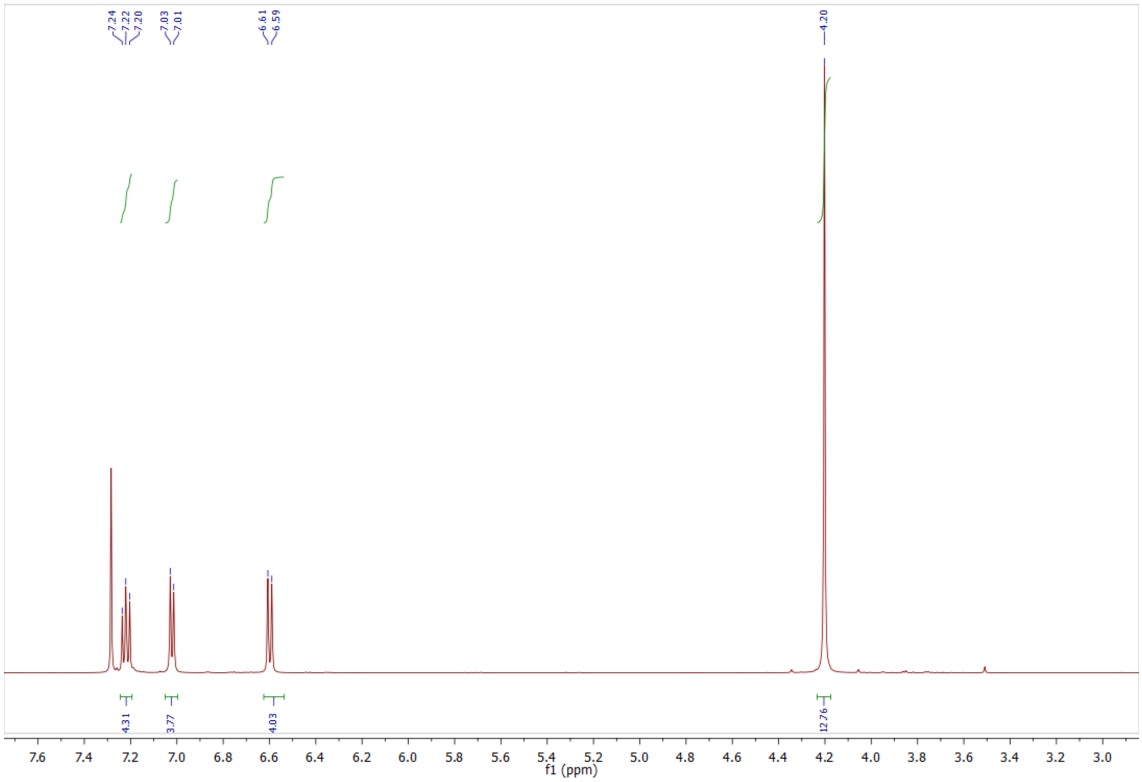

Figure S1: ^{1}H-NMR spectrum of **14** ($CDCl_3$)

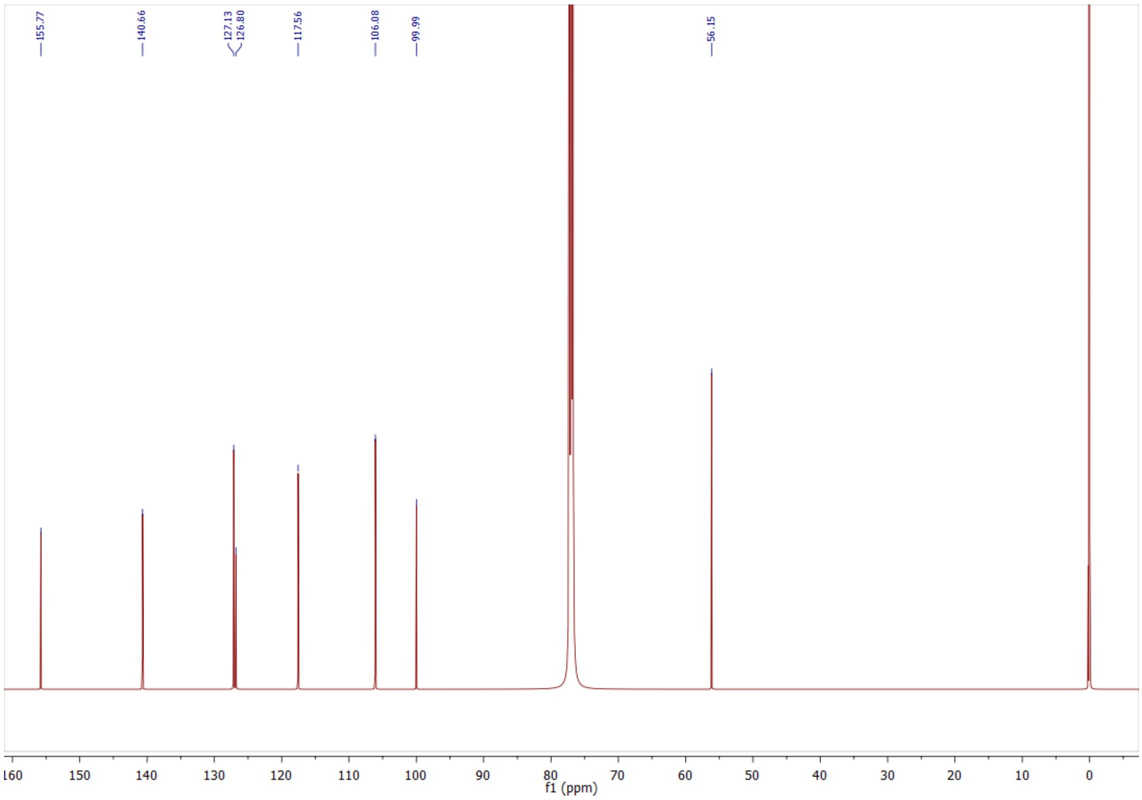

Figure S2: ^{13}C-NMR spectrum of **14** ($CDCl_3$)

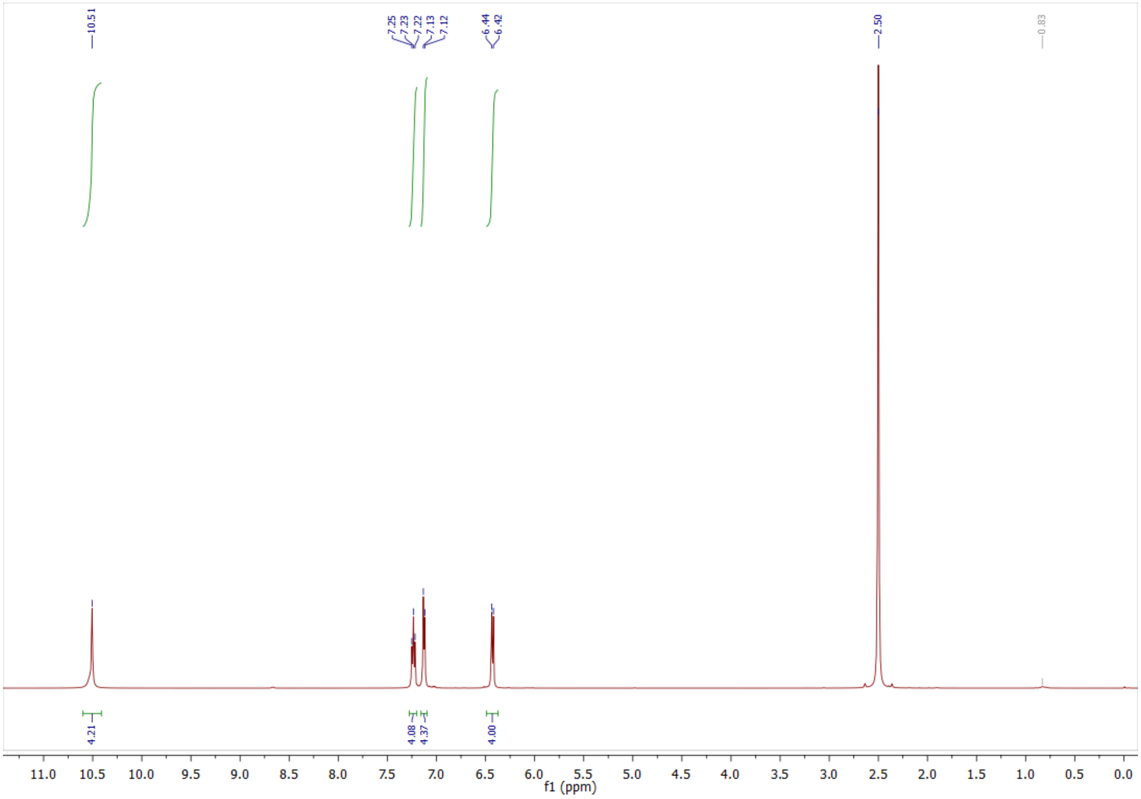

Figure S3: ^{1}H-NMR spectrum of **15** (DMSO-d_6)

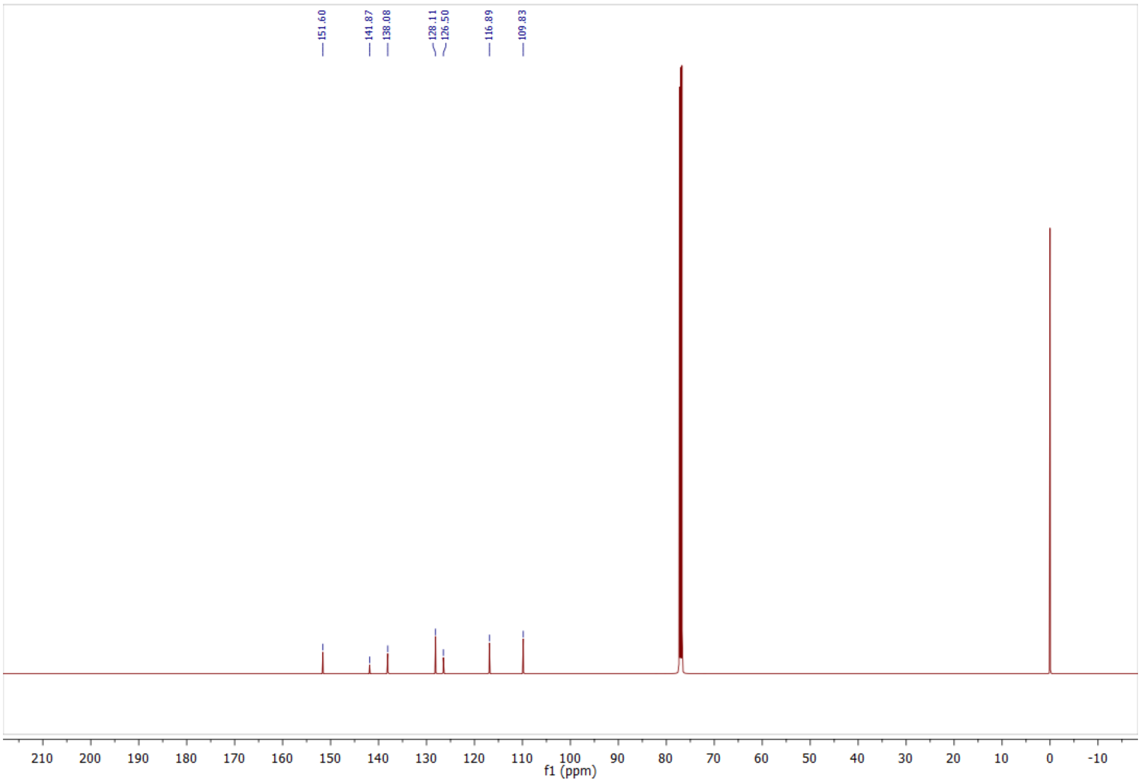

Figure S4: ^{13}C-NMR spectrum of **15** (DMSO-d_6)

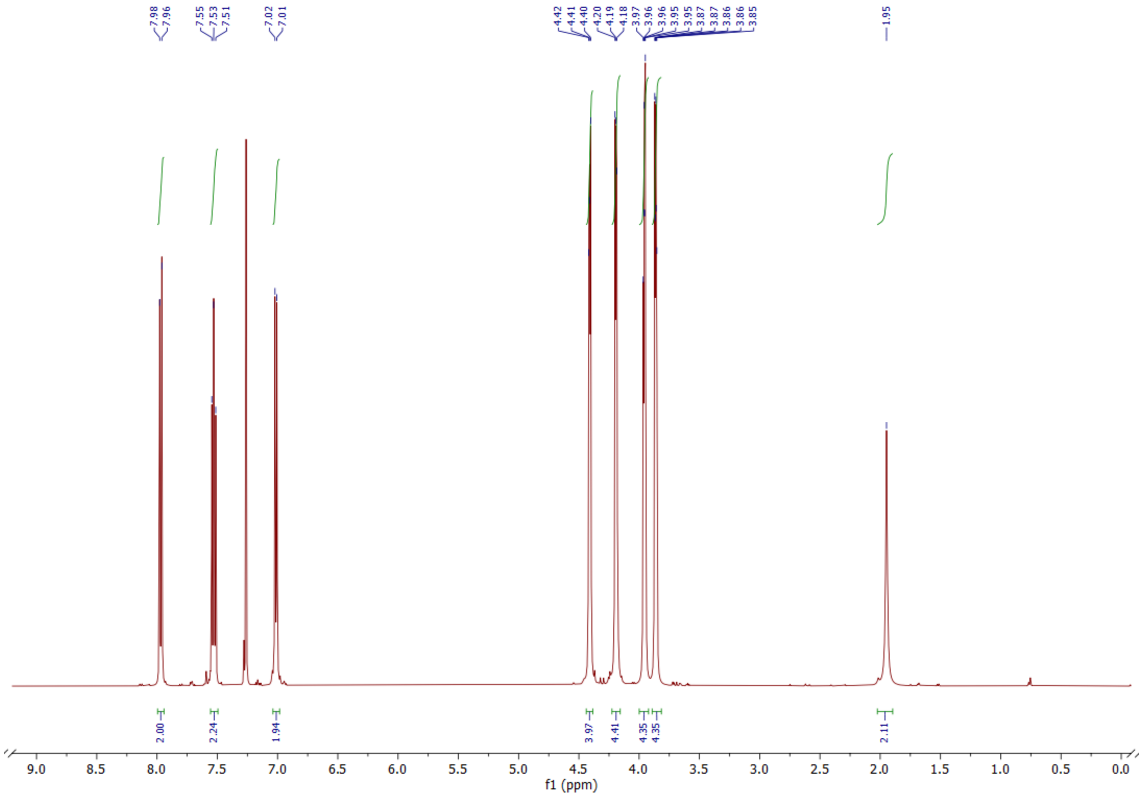

Figure S5: ^{1}H-NMR spectrum of **17** ($CDCl_3$)

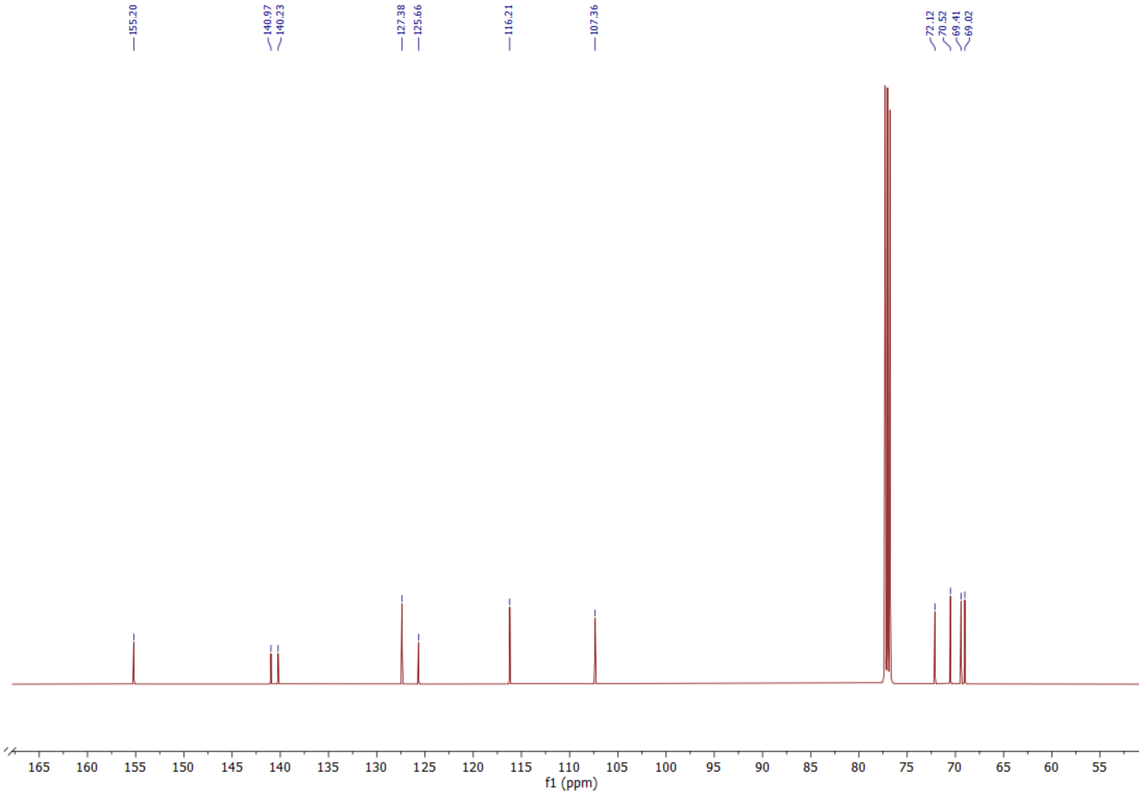

Figure S6: ^{13}C-NMR spectrum of **17** ($CDCl_3$)

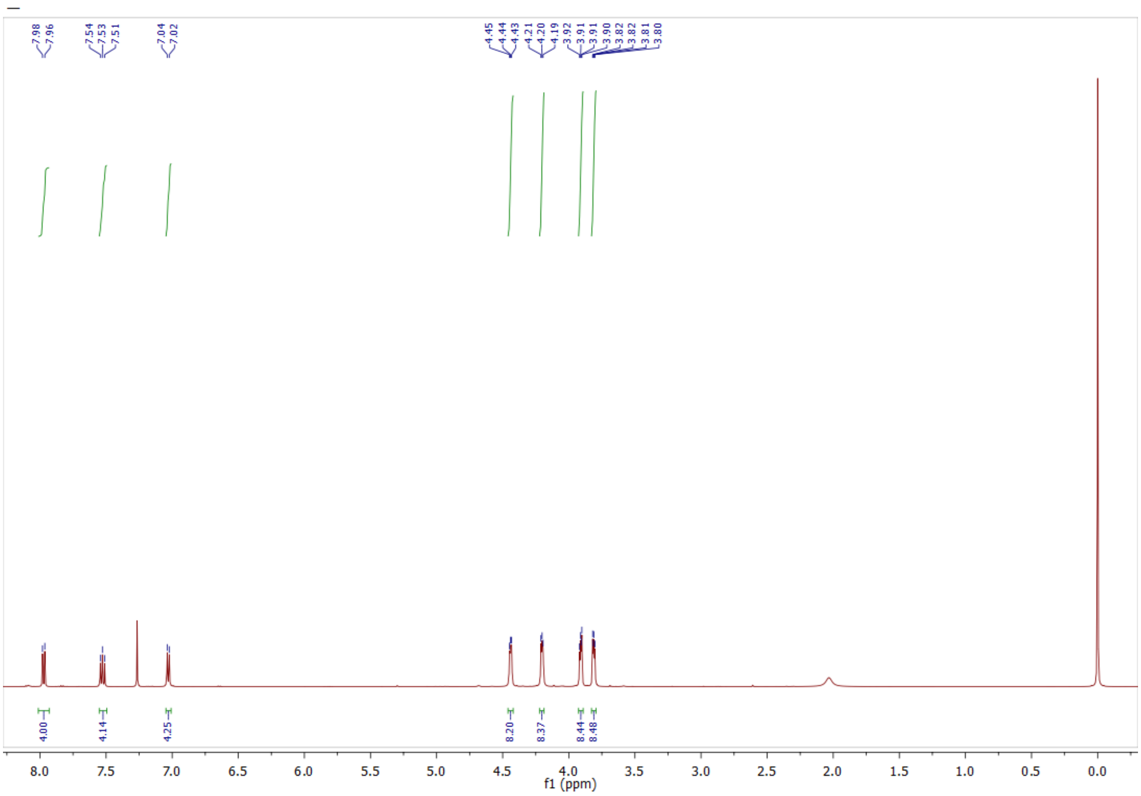

Figure S7: ^{1}H-NMR spectrum of **10** ($CDCl_3$)

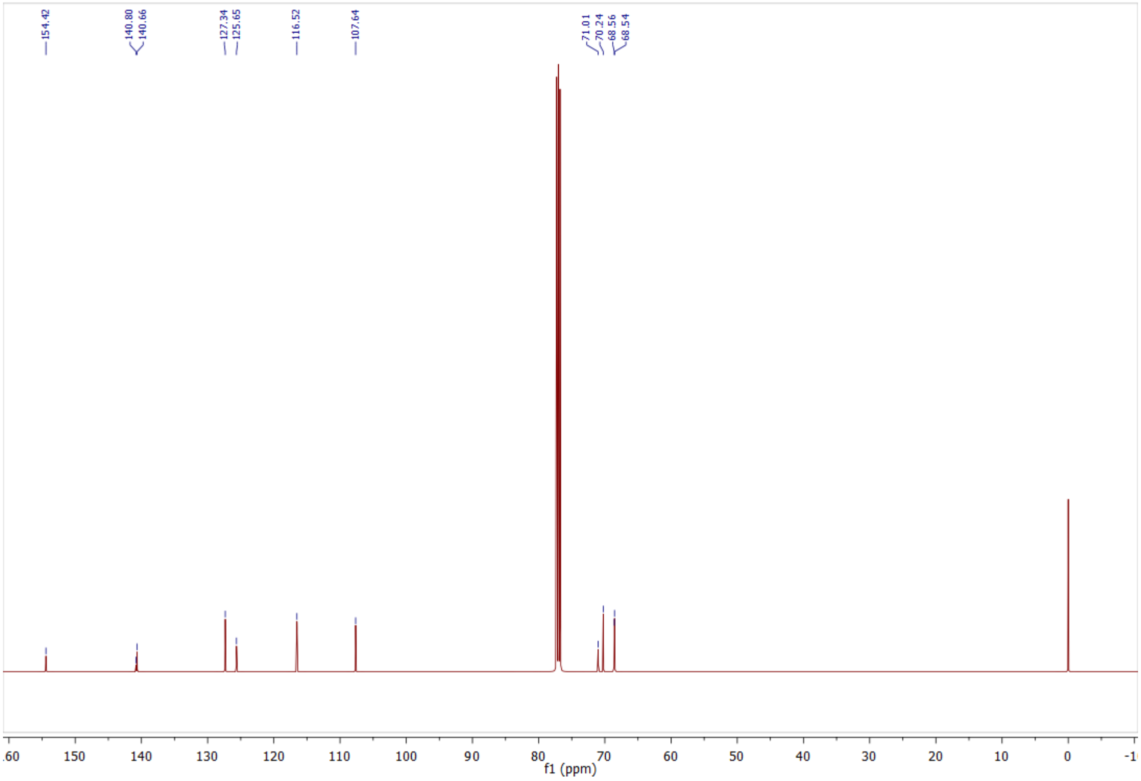

Figure S8: ^{13}C-NMR spectrum of **10** ($CDCl_3$)

2. UV-spectroscopic investigation of the new crown ether (**10**)

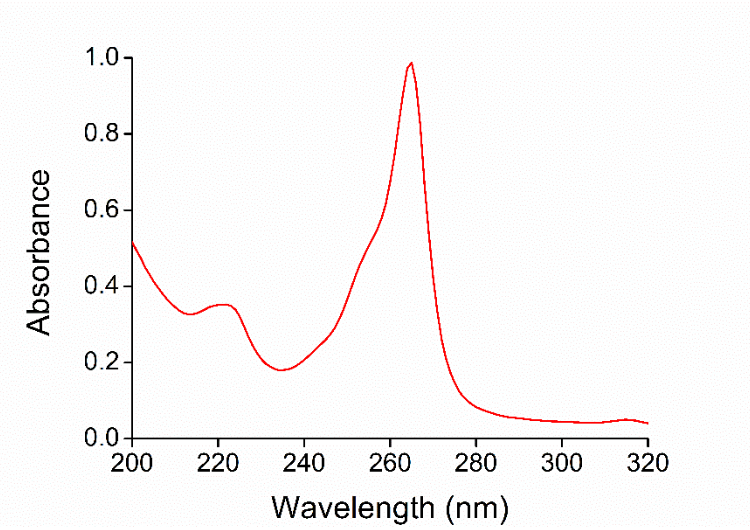

Figure S9: UV spectrum of **10** in acetonitrile

3. Results for neutral oligoamines

It is important to note, that the presence of water traces could not be fully excluded and the titration experiments were not carried out under an inert atmosphere. Although a partial protonation of the neutral guest molecules could take place, these experiments can serve as an estimation on the effects of replacing water with other solvents. It can be concluded, that the values of the stability constants for neutral amin analogues decreased at least one order of magnitude. However, investigating of these bioactive molecules in non-aqueous media is rarely relevant in practice.

Table S1. Calculated stability constants and cooperativity in binding for the determined consecutive 1:1 and 1:2 (host:guest) amine-coordination of the host molecule

**guest amine** [1]	**logK_1** [2]	**logK_2** [3]	**binding cooperativity effects** [4]
n-butylamine	1.5±0.2	<<1.5	non-cooperative
putrescine	1.7±0.2	<<1.7	non-cooperative
N,N'-bis(2-aminoethyl)-1,3-propanediamine	2.0±0.2	<<2.0	non-cooperative
norspermidine	2.8±0.2	<<2.0	non-cooperative
spermine	4.4±0.1	3.6±0.1	negative cooperativity (α=0.6)
spermidine	4.8±0.1	4.2±0.2	non-cooperative

[1] Added in acetonitrile solution in a neutral, free-amine form.

[2] Complex stability constant for 1:1 (host:guest) complexes as the first step of coordination.

[3] Complex stability constant for 1:2 (host:guest) complexes as the second step of coordination

[4] Calculated according to [54], where K_1=4K_2 refers to a non-cooperative stepwise binding, while α expresses the extent of cooperativity.

4. Reported data for the characterization of 9-chloro-4,5-dimethoxyacridine (**12**) [52,53]

R_f= 0.82 (silica gel TLC, dichloromethane:methanol = 10:1)

M.p.: 220–225°C (decomposition)

IR (KBr) ν_{max} = 3438, 3343, 3084, 3067, 3036, 3004, 2968, 2838, 1626, 1607, 1595, 1534, 1487, 1456, 1448, 1410, 1331, 1270, 1224, 1077, 971, 810, 746, 575 cm^{-1}

^{1}H-NMR (300 MHz, $CDCl_3$) δ = 4.14 (s, 6H, OCH_3), 7.06 (d, *J* = 8 Hz, 2H, Ar-H), 7.58 (t, *J* = 8 Hz, 2H, Ar-H), 7.99 (d, *J* = 8 Hz, 2H, Ar-H) ppm

^{13}C-NMR (75 MHz, $CDCl_3$) δ = 56.42, 106.84, 116.16, 125.86, 127.83, 140.67, 140.69, 155.66 ppm

HRMS m/z = 274.06292 $(M+H)^+$ (calculated for $C_{15}H_{12}ClNO_2$, 273.05566).
